# Quantitative Proteomics and Phosphoproteomics Analysis of Patient-Derived Ovarian Cancer Stem Cells

**DOI:** 10.1016/j.mcpro.2025.100965

**Published:** 2025-04-07

**Authors:** Giulia Franciosa, Valentina Nieddu, Chiara Battistini, Miriam Caffarini, Michela Lupia, Nicoletta Colombo, Nicola Fusco, Jesper V. Olsen, Ugo Cavallaro

**Affiliations:** 1 Novo Nordisk Foundation Center for Protein Research, Department of Cellular andMolecular Medicine, Faculty of Health and Medical Sciences, Copenhagen University, Copenhagen, Denmark; 2 Unit of Gynecological Oncology Research, European Institute of Oncology IRCSS, Milano, Italy; 3 Division of Gynecologic Oncology, European Institute of Oncology IRCCS, Milano, Italy; 4 Department of Medicine and Surgery, University of Milano-Bicocca, Monza, Italy; 5 Department of Pathology and Laboratory Medicine, European Institute of Oncology IRCCS, Milano, Italy; 6 Department of Oncology and Hemato-Oncology, University of Milan, Milano, Italy

**Keywords:** ovarian cancer, HGSOC, cancer stem cells, proteomics, phosphoproteomics

## Abstract

High-grade serous ovarian carcinoma (HGSOC) is the deadliest gynecologic cancer. Key to the progression and ultimate lethality of this subtype is the intra-tumoral heterogeneity, which is defined as the coexistence of different cell types and populations within a single tumor. Among those, ovarian cancer stem cells (OCSCs) are a distinct subpopulation of tumor cells endowed with stem-like properties, which can survive current standard therapies, resulting in tumor recurrence. Here, we generated *ex vivo* primary OCSC-enriched three-dimensional (3D) spheres from 10 distinct treatment naive patient-derived adherent (2D) cultures. We used state-of-the-art quantitative mass spectrometry to characterize the molecular events associated with OCSCs by analyzing their proteome and phosphoproteome. Our data revealed a stemness-related protein signature, shared within a heterogeneous patient cohort, which correlates with chemo-refractoriness in a clinical proteomics dataset. Moreover, we identified targetable deregulated kinases and aberrant PDGF receptor activation in OCSCs. Pharmacological inhibition of PDGFR in adherent OC cells reduced the stemness potential, measured by sphere formation assay. Overall, we provide a valuable resource to identify new OCSC markers and putative targets for OCSC-directed therapies.

## Highlights

• Mass spectrometry reveals a conserved stemness signature in ovarian cancer.
• Stemness proteins are involved in extracellular matrix remodeling and metabolism.
• The stemness signature correlates with chemo-refractoriness in clinical datasets.
• Aberrant PDGF receptor activation is a key feature of ovarian cancer stemness.
• PDGFR inhibition reduces stemness potential in patient-derived cancer cells.

## Graphical Abstract

**Figure:**
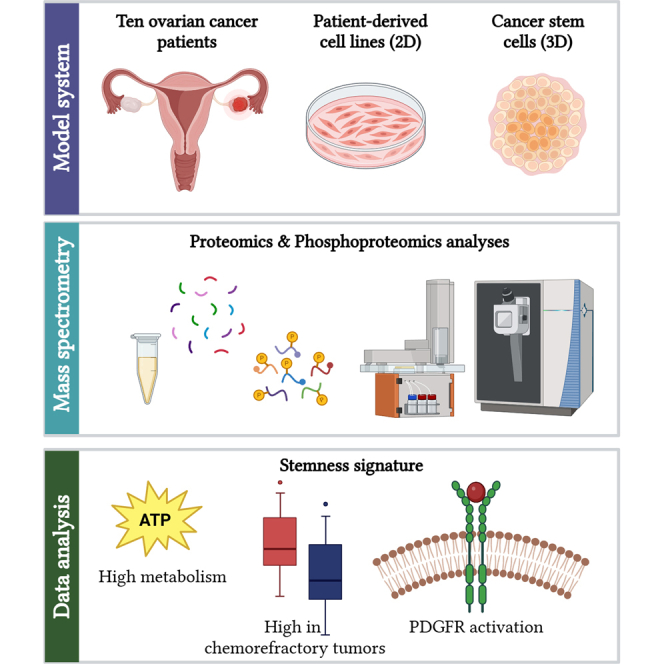

## In Brief

A quantitative mass spectrometry-based proteomics and phosphoproteomics analysis revealed a conserved protein signature of patient-derived ovarian cancer stem cells (OCSCs). This stemness-related signature correlated with chemo-refractoriness in a clinical proteomics dataset. Moreover, we found an aberrant activation of the PDGF receptor in OCSCs and showed that pharmacological inhibition of PDGFR reduced stemness potential. Our data unveiled new stemness-associated markers and pathways, as well as putative vulnerabilities for OCSCtargeted treatments.

Ovarian cancer (OC) comprises a heterogeneous group of malignancies caused by abnormal cell growth either in the ovaries, fallopian tubes or endometrium. Epithelial ovarian cancer (EOC) represents almost 90% of all cases, which are further classified into several subtypes. High-grade serous ovarian carcinoma (HGSOC) is the most common and lethal subtype of EOC; it arises from either the fallopian tube or the ovarian surface epithelium and accounts for the majority of ovarian cancer-related deaths, with a 5-years survival rate below 30% (1). The high mortality rate is mainly due to the lack of efficient tools for early diagnosis and to the lack of effective treatment options for advanced-stage disease. Therefore, understanding the molecular mechanisms of OC development and progression represents a critical step to improving patient survival.

Tumors are composed of heterogeneous cell populations. Among these, cancer stem cells (CSCs) represent a rare population of cells, derived from either the malignant transformation of a normal stem cell or the dedifferentiation of a mature cancer cell, that are responsible for giving rise to the whole tumor (2). The main features of CSCs are self-renewal, clonogenic potential, resistance to *anoikis*, quiescent state, acquisition of epithelial-mesenchymal transition (EMT) traits, ability to undergo differentiation through asymmetric cell division and resistance to chemotherapy. In addition, CSCs can generate a tumor upon transplantation into a recipient organism. Based on this function, CSCs are also commonly called tumor-initiating cells (TICs) (3). The role of CSCs in tumor initiation and recurrence, as well as chemoresistance, makes them the ideal drug target for tumor eradication.

Proteomics is the large-scale analysis of proteins in a biological sample (4) and has been previously employed to study OC biology. The Clinical Proteomic Tumor Analysis Consortium (CPTAC) thoroughly investigated the impact of genomic alterations on cancer biology at a functional level by comprehensively analyzing a large number of HGSOC specimens by mass spectrometry (MS)-based proteomics, glycoproteomics and phosphoproteomics (5, 6, 7, 8). However, these studies employed bulk tumor samples, losing cell identity information. Laser-capture microdissection combined with MS-based proteomics has been successfully used to separately analyze tumor cells and stroma in HGSOC (9). Still, information about proteome changes between differentiated tumor cells and their stem cell counterparts is not yet available.

Biological properties of CSCs are intrinsically difficult to assess within the original tumor, due to the lack of general and robust markers. Sphere formation under non-adherent conditions is a cell-based assay, which allows enrichment for CSCs as it exploits their ability to resist *anoikis* and generate clonal spheroidal colonies (10). To characterize the molecular traits of CSCs from HGSOC (ovarian CSCs, OCSCs), in this study we generated patient-derived primary cultures from HGSOC peritoneal ascites, which are known to be enriched in tumor cells with stem-like properties, as tumor cells in this location survive and proliferate as spheres in the absence of adhesion (11, 12). We performed quantitative MS-based proteomics and phosphoproteomics on cells either grown as adherent two-dimensional (2D) cultures or as non-adherent stem-cell enriched three-dimensional (3D) spheres. We showed that OCSCs exhibit homogeneous proteome and phosphoproteome signatures, which can potentially be exploited to define specific molecular markers and develop OCSC-targeted therapies.

## Experimental Procedures

### Primary Cells

All ascites samples were obtained upon informed consent from women undergoing surgery at the Gynecology Division of the European Institute of Oncology and collected *via* standardized operative procedures approved by the Institutional Ethical Board (European Institute of Oncology). Tumor histology was confirmed by a board-certified pathologist (G. Bertalot), while the identity of cancer cells was confirmed by immunostaining for cytokeratins 5, 7 and 8, or pan-cytokeratins. The purity of primary cell culture was consistently over 95%. Tissue isolation and culture conditions of primary cells were performed as described previously (13). This study was conducted in compliance with the principles of the Declaration of Helsinki.

### Sphere Formation Assay

Sphere formation assays were performed as described previously (14). Briefly, single-cell suspensions were obtained from trypsinization of primary cultures and seeded at low density (5000 cells/ml) under non-adherent conditions in poly-(2-hydroxyethyl methacrylate)-coated dishes (Sigma-Aldrich, cat# P3932) and allowed to form clonal spheres. CSC-enriched sphere were maintained in MEBM (Lonza, cat# CC-3151), supplemented with 2% B27 (Thermo Fisher Scientific, cat# 17504044;), 2 mM L-glutamine, 100 U/ml penicillin, 100 μg/ml streptomycin, 5 μg/ml insulin (Thermo Fisher Scientific, cat# RP10935), 0.5 μg/ml hydrocortisone (Sigma-Aldrich, cat# H0888), 1 U/ml heparin (Voden, cat # 07980), 20 ng/ml epidermal growth factor (EGF, Merck, cat# E4127) and 20 ng/ml fibroblast growth factor-2 (FGF2; Peprotech, cat# AF-100-18B).

Sphere formation was assessed 5 to 10 days after seeding. Spheres were then dissociated with StemPro Accutase (Thermo Fisher Scientific, cat# A1110501), according to the manufacturer's protocol, and re-plated under the same conditions for 72 h to obtain second-generation spheres, which were then used for MS analysis.

PDGFR inhibitors were purchased from Merck Life Science (Axitinib: cat. No. PZ0193; Imatinib Mesylate: cat. No. SML1027). The inhibitor was added when seeding the cells for the second-generation spheres and maintained for the entire duration of the sphere formation assay (72 h).

### Immunoblotting

Spheres and 2D cells were lysed in hot lysis buffer (2.5% SDS, 125 mM Tris-HCl [pH 6.8]), after 15 min incubation at 95 °C.

Protein concentration was determined using a Pierce BCA Protein Assay kit (Thermo Fisher Scientific, Inc, cat# 23227) according to the manufacturer's instructions. Equal amounts of protein extracts (20 μg) were resolved in acrylamide gel and transferred onto nitrocellulose membranes. The membranes were incubated overnight at 4 °C with the following primary antibodies: GPNMB (LSBio, cat# LS-B11132), CHI3L1 (R&D Systems, cat# AF2599), phospho-PDGFR beta tyrosine-1009 (Cell Signaling Technology, cat# 3124), PDGFR beta (Cell Signaling Technology cat# #4564), GAPDH (Sigma-Aldrich, cat# ABS16), and vinculin (Sigma-Aldrich, cat# V9131). Membranes were incubated with HRP-conjugated secondary antibody (Bio-Rad Laboratories) for 1 h at room temperature. The signal was detected by the Clarity Western ECL Substrate (Bio-Rad, cat# 1705062) following the manufacturer's protocol and the images were acquired using ChemiDoc (Bio-Rad).

### RNA Extraction and qRT-PCR

Total RNA was extracted using the RNeasy Mini Kit (QIAGEN, cat# 217004) according to the manufacturer's protocol and quantified using the Nanodrop instrument (Thermo Fisher Scientific). Preparation of cDNA and qRT-PCR was performed by the Cogentech qPCR Service (Milan, Italy). Gene expression levels for each gene were analyzed and normalized against the housekeeping genes GAPDH and HPRT1. TaqMan assays for specific genes are listed in the table below. Normalized expression changes were determined with the comparative threshold cycle (2−ΔΔCT) method.

**Table:** 

Gene	Taqman assay
*NANOG*	hs02387400_g1
*KLF4*	hs00358836_m1
*SOX2*	Hs01053049_s1
*POUF51*	Hs04260367_gh
*NT5E*	Hs00159686_m1
*CDH1*	hs00170423_m1
*CDH2*	hs00169953_m1
*FN1*	hs00365052_m1
*SNAI1*	hs00195591_m1
*SNAI2*	hs00950344_m1
*TWIST1*	hs00361186_m1
*VIM*	hs00185584_m1
*ZEB1*	hs00232783_m1
*GAPDH*	Hs99999905_m1
*HPRT1*	hs99999909_m1

### Immunofluorescence

Cultured adherent cells or cytospins with spheres were fixed with 4% paraformaldehyde for 5 min at room temperature and then permeabilized in ice-cold PBS, 0.5% Triton X-100 for 3 min at 4  °C. After blocking for 1 h at room temperature with blocking buffer (PBS, 0.2% BSA, 1% donkey serum, 0.05% Tween-20 and 0.02% NaN3), cells were incubated for 2 h with the following primary antibodies, diluted in blocking buffer: anti-CD73 (clone AA60E3.3), generated by E. Dejana (Milan) and produced by the Biochemistry Unit at Cogentech, Milan; anti-CD24 (BD Biosciences, catalog no. 555427); anti-CD106 (BD Biosciences, catalog no. 555647); anti-CD44 (clone Hermes-3, from U. Günthert, Basel).

Cells were then washed with PBS and incubated with the Alexa Fluor-conjugated secondary antibodies (Jackson Laboratories) for 1 h at room temperature. Nuclei were counterstained with DAPI (Sigma-Aldrich, cat# 32670). Images were acquired using the Leica SP8 Confocal microscope.

### Cell Lysis, Protein Extraction and Digestion for Mass Spectrometry

Cells were lysed with boiling guanidine-hydrochloride lysis buffer (6M Gnd-HCl, 100 mM Tris-HCl pH 8.5, 5 TCEP, 10 mM CAA). Lysates were heated at 95 °C for 10 min, while shaking. Samples were diluted with 25 mM Tris-HCl pH 8.5 to reach a Gnd-HCl concentration of 1M and sonicated (Vibra-Cell VCX130, Sonics) for 2 min, with pulses of 1 s on and 1 s off. Protein concentration was determined using the Bradford assay (Bio-Rad). Proteins were pre-digested with endoproteinase Lys-C (Wako) for 2 h at room temperature in an enzyme/protein ratio of 1:100 before overnight digestion with trypsin (Sigma-Aldrich) at 37 °C in an enzyme/protein ratio of 1:50. After overnight digestion, enzymatic activity was quenched by acidifying the lysates using trifluoroacetic acid (TFA) at a final concentration of 1% and ensuring the pH of the samples being around 2. Precipitates were removed by centrifugation at 3000*g* for 5 min.

750 ng of digested peptides for single-shot proteome analysis were loaded on C18 Evotips (Evosep) for MS analysis. Two technical replicates per sample were prepared.

Digested peptides for TMT labeling were purified and concentrated on reversed-phase C18 Sep-Pak cartridges (Waters). After elution with 40% acetonitrile (ACN) followed by 60% ACN, a SpeedVac concentrator (ThermoFisher Scientific), operating at 60 °C, was utilized to concentrate the samples. Peptide concentration was estimated by measuring absorbance at 280 nm (A280) on a NanoDrop spectrophotometer (ThermoFisher Scientific).

### TMT labeling

For TMT-labeling (11-plex kit, Thermo Fisher Scientific) of samples, 28 μg of peptides per sample were used. A pooled sample for normalization between TMTs was prepared by combining 2.8 μg of peptide from each individual sample. Labeling was performed according to the manufacturer's instructions. All samples were adjusted to a concentration of 50% ACN and alkalized to a pH of 8 using HEPES, for a final concentration of 30 mM. The labeling reaction between the peptides and the TMT reagents was conducted over 1 h using 3 μl TMT reagent per sample. The reaction was then quenched for 15 min using 1% hydroxylamine (1:1 hydroxylamine:TMT), after which the samples were pulled, acidified and ACN was then evaporated in the SpeedVac concentrator. TMT labeled peptides were purified on reversed-phase C18 Sep-Pak cartridges (Waters). After elution with 40% acetonitrile (ACN) followed by 60% and 80% ACN, the SpeedVac concentrator was used to concentrate the samples to reach a volume below 25 ul.

Patients 1 to 5 comprised the first TMT-plex, and patients 6 to 10 comprised the second TMT-plex. The 11th channel 131C is the reference channel and is made by a pool of all 20 samples (see supplemental Table S7 for the detailed TMT labeling scheme).

### Enrichment of Phosphorylated Peptides

Ti-IMAC phosphopeptide enrichment was carried out on a KingFisher Flex robot (Thermo Fisher Scientific) (15) in 96-well format, as previously described (16). 308 μg of peptide were used for enrichments, with 30 μl of magnetic Ti-IMAC HP beads (ReSyn Biosciences). The 96-well comb is stored in plate #1, Ti-IMAC HP beads in 100% ACN in plate #2 and loading buffer (1 M glycolic acid, 80% ACN, 5% TFA) in plate #3. The sample is mixed with loading buffer and added in plate #4. Plates 5 to 7 are filled with 500 μl of washing solutions (loading buffer, 80% ACN, 5% TFA, and 10% ACN, 0.2% TFA, respectively). Plate #8 contains 200 μl of 1% NH4OH for elution. The beads are washed in loading buffer for 5 min at medium mixing speed, followed by binding of the phosphopeptides for 20 min and medium speed. The sequential washes are performed in 2 min and fast speed. Phosphopeptides are eluted in 10 min at medium mixing speed.

### High-pH Reversed-phase Stage-Tip Fractionation of Phosphopeptides

Labeled phosphopeptides were manually fractionated using high-pH reversed-phase chromatography in stage-tips, as previously described (16). In-house stage tips were prepared using low-bind 200 μl tips with three discs of 3M Empore C18 (Waters). Stage tips were conditioned with 50 μl methanol, 50 μl of 20 mM ammonia in ACN, 2 × 100 μl 20 mM ammonia in water. Phosphopeptides were loaded 3 times on the tip. 50 μl of 20 mM ammonia in water was loaded on the tip and combined with the flow through (FT). The peptides were eluted successively with 4, 8, 12, 20, 80% ACN with 20 mM ammonia. The two last fractions were pooled together. ACN was evaporated and the samples acidified with formic acid (pH < 3) before being loaded on C18 Evotips (Evosep) for MS analysis.

### Mass Spectrometry Analysis

Samples were analyzed on the Evosep One LC system (17) coupled to an Orbitrap Exploris 480 (18). Samples were separated on an in-house packed 15 cm analytical column (150 μm inner diameter), packed with 1.9 μm C18 beads, and the column temperature was maintained at 60 °C using an integrated column oven (PRSO-V1, Sonation GmbH). Pre-programmed gradients were used: 30 samples per day for proteome (SPD), and 60 SPD for phosphoproteome.

The mass spectrometer was operated in positive ion mode, with spray voltage at 2 kV, heated capillary temperature at 275 °C, and funnel RF frequency at 40. The Peptide match was set to off, and isotope exclusion was on. We used data-independent acquisition (DIA) for label-free total proteome and data-dependent acquisition mode (DDA) for TMT-labeled samples, respectively.

For DIA, full MS resolution was set to 120,000 at m/z 200 and normalized AGC target was set at 300%, with an injection time of 45 ms, and the precursor scan range was set to 361 to 1033 m/z. The MS/MS acquisition was set to 15,000 resolution, and injection time to 22 ms; normalized collision energy was set at 27%; normalized AGC target value was set at 1000%; 49 windows of 13.7 m/z were used with an overlap of 1 Da.

For DDA, full MS resolution was set to 60,000 at m/z 200 and normalized AGC target was set at 300%, with an injection time of 25 ms, and the precursor scan range was set to 350 to 1400 m/z. The MS/MS acquisition was set to 15,000 resolution, and injection time to 22 ms; normalized collision energy was set at 35%; normalized AGC target value was set at 200%; isolation window was set at 0.8 m/z; dynamic exclusion was set at 30 s; intensity threshold was set at 2E5; a Top12 method was used; the turbo-TMT (centroid mode) was enabled (19).

### Mass Spectrometry Raw Data Processing

DIA data were analyzed on Spectronaut (20) version 18 in directDIA mode with the standard settings. The Human Uniprot fasta file (downloaded in March 2023; 20,422 entries) was supplemented with a contaminant fasta file containing 370 entries (21), downloaded from:

https://github.com/HaoGroup-ProtContLib. Carbamidomethyl was set as a fixed modification, and N-terminal acetylation and oxidation of methionine as variable modifications. Up to five variable modifications were allowed. Trypsin was set as cleavage enzyme with a maximum of two missed cleavages. The mass tolerance was set to dynamic in both MS1 and MS2 level with a correction factor of 1. A Q value of 1% against mutated decoys was applied to filter identifications with a false-discovery rate level of 0.01 at both the peptide and protein level. Quantification was performed using the automatic setting, which was Quant 2.0 in this Spectronaut version. Cross-run normalization and imputation were not enabled, with the exception of the PDGFRB precursor analysis (supplemental Fig. S14*C*).

TMT phosphoproteome raw MS data were analyzed using the MaxQuant software (22), version 2.6.6.0, with the integrated Andromeda search engine (23). Files were searched against a target/decoy version of the human UniProt database (downloaded in May 2024; 20,434 entries). The UniProt database version was supplemented with the MaxQuant contaminant list. Carbamidomethyl was set as a fixed modification; oxidation of methionine, N-terminal acetylation, deamidation of asparagine and glutamine, and phosphorylation of serine, threonine, and tyrosine were included as variable modifications. A maximum of four variable modifications and two miscleavages were allowed per peptide. The precursor mass tolerance was set to 20 ppm, and the initial fragment mass tolerance was set to 0.6 Da. A false discovery rate of 1% was applied for peptide, protein and site identifications. TMT correction factors were not used. Channel 11 was set as the reference channel. Normalization was set to “weighted ratio to reference channel”. The isobaric weight exponent was set to 0.75 (standard setting). Match between runs was enabled (24) with the standard settings, with the only exception of the matching time window that was set to 0.7 min.

To calculate TMT labeling efficiency, TMT126C was set as a variable modification, both at lysine residues and the N-terminal, together with phosphorylation of serine, threonine, and tyrosine. Carbamidomethyl was set as a fixed modification. A maximum of five variable modifications and one miscleavage were allowed per peptide.

### Bioinformatic Analysis

Data analysis was done using the R software package v4.4.1 (with RStudio v2024.09.0) and the Perseus Software (25) v1.6.15. The majority of plots were performed using the “ggplot2” R package. Heatmaps were performed using the “pheatmap” R package and the clustering was performed using Euclidean distance, unless stated otherwise.

#### Proteome Data

Protein groups' MS intensities were log2-transformed to stabilize the variance. Data were filtered by removing potential contaminants and entries without gene names. Data normalization was performed by median realignment: the median of all proteins of a run was subtracted from each protein intensity of the corresponding run; then, the mean of the 40 medians was summed to each value. Afterward, technical replicates (2 MS injections per sample) were averaged.

Principal component analysis was performed after removing all missing values; data were centered but not scaled. The PCA plot were performed through the “ggbiplot” R package.

We considered a protein as unique to OCSCs if it had no values in all patients (=10) in the bulk condition, but had at least four valid values in OCSCs. Conversely, we considered a protein as unique to the bulk condition if it had no values in all patients (=10) in the OCSCs, but had at least four valid values in the bulk condition.

Before differential expression analysis, data were filtered by requiring at least three valid values per group (bulk and OCSCs). Differential expression analysis was performed by using the "Limma" R package (26), accounting for paired measurements (blocking on the patient). *p* values were corrected by using the Benjamini–Hochberg (BH) procedure. The fold-change was calculated as mean(protein X)_OCSC_ - mean(protein X)_bulk_ in log2 space. A fold-change cutoff was calculated separately for up- and down-regulated proteins as ±2× standard deviation of fold-changes. The calculated fold-change cutoff was equal to 0.89 for up- and −0.7 for down-regulated proteins. Proteins were considered significantly regulated if their adjusted *p* value was ≤ 0.01 and their fold-change exceeded the calculated fold-change cutoff.

Gene ontology (GO) and pathway enrichment analyses were performed against the whole dataset as background. The input protein list included all up-regulated/down-regulated proteins as defined above plus all proteins unique to OCSCs/bulk, respectively. GO enrichment was performed with the “topGO” R package (27) with the ontologies biological processes (BP) and cellular components (CC). Fisher's exact test with the “weight01” algorithm was used. Node size was set to five for BP and to 10 for CC. *p* values were corrected by using the BH procedure. KEGG and Reactome pathway enrichment analyses were performed with the R packages “clusterProfiler” (28) and “ReactomePA” (29). Gene set enrichment analysis (GSEA) (30) was performed through clusterProfiler by using the molecular signature database (MSigDB) (31, 32). Proteins were ranked by fold-change OCSCs vs bulk.

Functional protein networks displayed were generated through STRING (33) and the “Omics Visualizer” app for Cytoscape (34). Proteins used for this analysis belong to the GO terms highlighted in blue in supplemental Figs. S7 and S8 and are listed in supplemental Table S8.

Hierarchical clustering analysis of fold-changes OCSCs vs bulk was performed using Euclidean distance to define clusters of patients. To find proteins with significantly different fold-change between the two clusters, we applied the Mann-Whitney U Test. Proteins with a *p* value < 0.02 were considered for further analysis.

#### Processing of Proteomics Data From Other Publications for Single-Sample Gene Set Enrichment Analysis (ssGSEA)

Proteome data from Yang *et al*. (35) and Chowdhury *et al*. (8) were downloaded from the respective publications.

In the dataset from Yang *et al*., murine gene IDs were converted to human IDs using the R package “homologene”. Proteins without a human homolog were discarded. Intensities of proteins with the same gene ID were averaged. Pooled samples were removed. Proteins with more than 30% of missing values across the dataset were removed. Log2 intensities were normalized by median realignment through the R package "DAPAR". Missing values were considered missing not at random (MNAR) and imputed using QRILC from the R package “imputeLCMD”.

In the dataset from Chowdhury *et al*., patient samples analyzed multiple times in different TMT plexes were averaged.

Gene sets for ssGSEA were created using significantly regulated proteins and proteins completely missing in one condition (attached in txt format as supplemental Table S9).

ssGSEA was performed on Gene Pattern (36) with default settings.

#### Phosphoproteome Data

Enrichment and labeling efficiency per TMT-plex were calculated by using the MaxQuant table “modificationSpecificPeptides”.

The “Phospho (STY)Sites” table from MaxQuant was used for bioinformatic analysis, after applying the “expand the site table” function implemented in Perseus. MS intensities were Log2-transformed and filtered by removing reverse hits, potential contaminants, rows without a gene name and rows without any quantification value. Phosphorylation sites with a localization score <0.75 were excluded. MS reporter intensities were normalized by quantile-based normalization, performed through the "Limma" R package (37).

Differential expression analysis was performed as discussed above for the proteome data. Fold-change cutoffs were equal to 0.84 for up- and −0.71 for down-regulated proteins.

Kinase enrichment analysis was performed by using the RoKAI web tool (https://rokai.io) (38), version 2.3.0. As input, the logFC calculated by Limma was used. Values from the same site and different multiplicities were averaged. The fold-change was set on “raw” and phosphatases were not included. Kinases were considered regulated if the *p* value was ≤0.05 and the substrates were ≥3.

Normalization on the proteome level was performed by subtracting the protein intensity from the corresponding phosphorylation sites (in log2 space). Both datasets were first normalized as described above.

#### Kaplan-Meier Survival Curves

Kaplan-Meier survival curves were performed through the web-based tool KM plotter (https://kmplot.com) (39), using mRNA gene chip data for ovarian cancer.

### Experimental Design and Statistical Rationale

In this study, different individuals are considered as biological replicates. The number of biological replicates (=10) was set based on the availability of appropriate samples in the biobank. For the proteome analysis, technical replicates were acquired (the same sample was injected twice in the MS). These were averaged before statistical analysis. Since the aim of this study was to identify differences between spheres and bulk, a paired design was employed (blocking on the individual).

## Results

### Characterization of Patient-Derived Ovarian Cancer Stem Cells by Quantitative MS-Based Proteomics

**Fig. 1:**
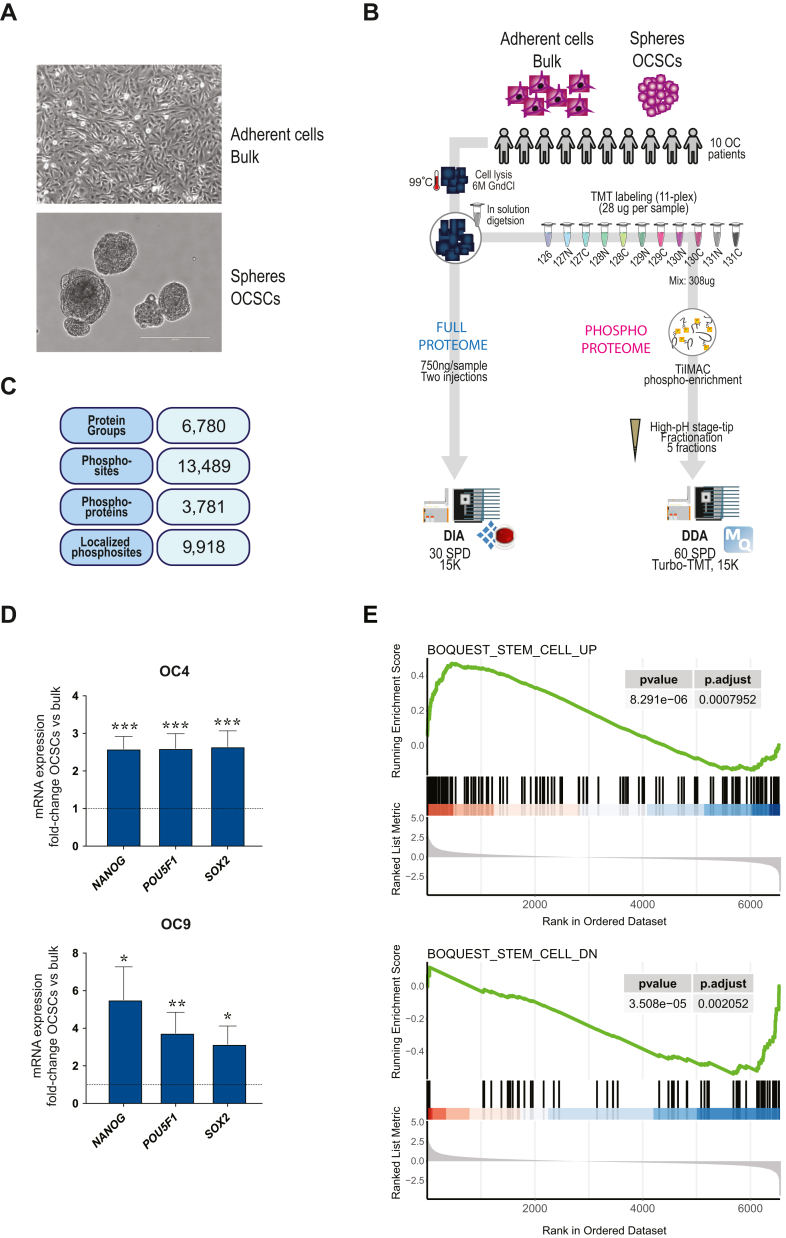
**Proteomic profiling of a cancer stem cell model in ovarian cancer**. *A*, representative images of primary tumor cells from an HGSOC patient, cultured for 7 days either as 2D, adherent cells (*left*) or as 3D, spheres (*right*). Scale bar, 200 μm. *B*, experimental workflow employed in this study. *C*, number of IDs obtained in this study. *D*, mRNA expression of stem-associated genes in OCSCs normalized on their adherent counterpart (*dashed line*) in two patient-derived cultures. Comparisons between experimental groups were performed by unpaired, two-sided, two-sample Student's *t* test. ∗ = *p* < 0.05; ∗∗ = *p* < 0.01; ∗∗∗ = *p* < 0.005. *E*, gene set enrichment analysis (GSEA) using the curated gene sets CGP (chemical and genetic perturbations). Proteins were ranked by Log2 fold-change OCSCs/bulk. DDA = data-dependent acquisition; DIA: data-independent acquisition; GndCl = guanidinium chloride; SPD = samples per day; TiIMAC = titanium IMAC; TMT = tandem mass tags.

To characterize the proteome and phosphoproteome of OCSCs, we employed state-of-the-art MS technology. The study included 10 primary HGSOC cultures, obtained from ascites of treatment-naive ovarian cancer patients (supplemental Table S1), which were generated in adherent (2D) conditions. From each 2D culture (bulk), matched OCSC-enriched cultures were obtained by the sphere formation technology (see Experimental Procedures) (Fig. 1*A*). Samples for global proteome analysis were acquired without prior labeling (label-free) in data-independent acquisition (DIA) mode. Due to the low amount of input material, samples for phosphoproteomics were labeled with tandem mass tags (TMTs) and multiplexed, prior to titanium immobilized metal affinity chromatography (Ti-IMAC) and MS analysis. In conjunction with TMT labeling, the phosphoproteomics workflow used offline high-pH reverse-phase stage-tip fractionation prior to online liquid chromatography tandem mass spectrometry (LC-MS/MS) to provide a broad coverage for phosphopeptide identification and quantification (16) (Fig. 1*B*). A total of 6780 protein groups were identified with high confidence (protein-level false discovery rate [FDR] < 1%) in the global proteomic experiment, with a mean of 6326 in the adherent condition and 6202 in the spheroid condition (Fig. 1*C*, supplemental Fig. S1*A* and supplemental Table S2). In the phosphoproteomic experiment, we identified and quantified 13,489 phosphosites, around 10,000 per TMT-plex (supplemental Fig. S1*B*), on 3781 protein coding genes (Fig. 1*C* and supplemental Table S3). Of those sites, 9918 (∼8000 per TMT-plex) were localized with a high confidence (localization score above 0.75) (Fig. 1*C* and supplemental Fig. S1*B*). Six thousand eight hundred forty-five localized phosphosites were shared between TMT-plexes, while 1397 were unique to TMT1 and 1676 were unique to TMT2 (supplemental Fig. S1*C*). The phosphopeptide capture efficiency was 87% for TMT1 and 92% for TMT2. The TMT labeling efficiency for phosphorylated peptides was 97% in both TMT-plexes.

### Stemness of OC Spheres

To confirm the enrichment of OCSCs in spheres compared to bulk cultures, we performed qRT-PCR on a subset of patients (number 4 and 9) for the established stemness markers NANOG, POUF51 and SOX2 (14) (Fig. 1*D*). Both models showed upregulation of all three markers in spheres compared to bulk, confirming that the cells in spheres are stem cells in nature. Moreover, we confirmed the purity of the stem cells' population derived through sphere formation assay by performing immunofluorescence staining in two additional patients (number 13 and 14) not included in the proteomics study. Three previously defined stemness markers (CD73/NT5E, CD106/VCAM1 and CD44) (14) were expressed highly homogeneously in spheres, while the negative marker CD24 showed no expression (supplemental Fig. S2).

We also confirmed the up-regulation of stemness-associated genes in spheres on a global level by performing gene set enrichment analysis (GSEA) using the curated gene sets CGP (chemical and genetic perturbations; supplemental Table S5). This analysis underlined the significant enrichment of stemness genes in spheres and the enrichment of non-stemness genes in adherent cells (Fig. 1*E*).

### Comparison of Protein Abundance Between OCSCs and Bulk OC Cells

**Fig. 2:**
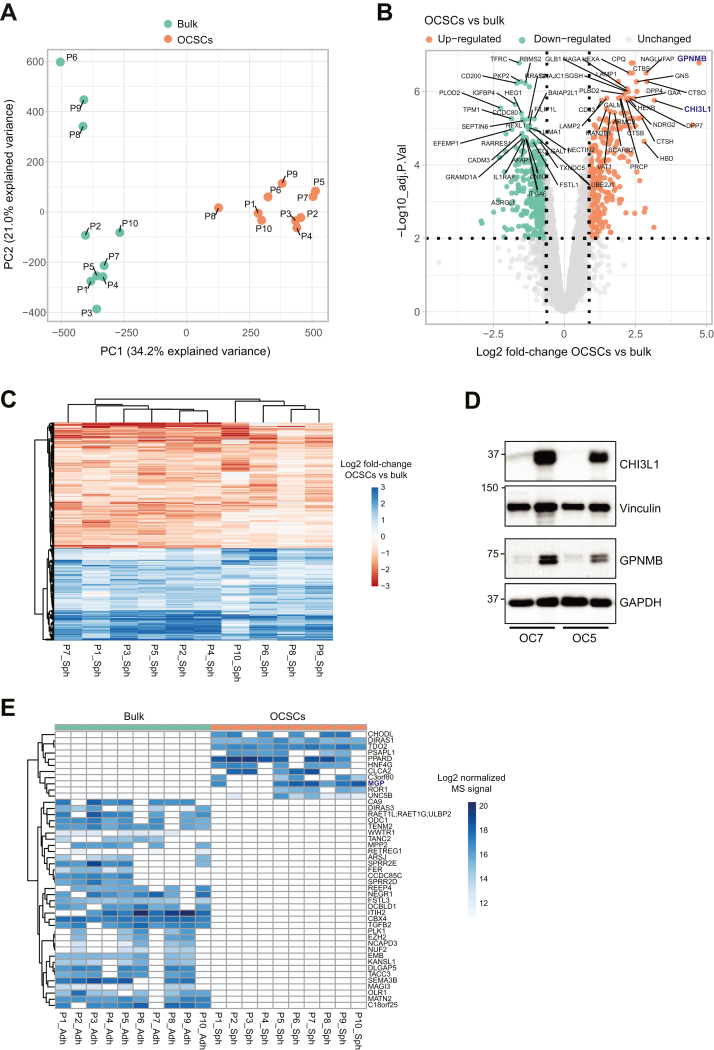
**Proteomic analysis of ovarian cancer stem cells**. *A*, principal component analysis (PCA) of the proteomic data. *B*, Volcano plot analysis of the proteomic data. Proteins colored in *orange* or *green* are considered *up-* or *down*-regulated, respectively. Proteins were ranked by Limma t statistics, and the for the *top* 30 on each side the gene name is displayed. *C*, heatmap of log2 fold-changes OCSCs vs bulk for significantly regulated proteins. *D*, immunoblot analysis for GPNMB and CHI3L1 in whole cell lysates from two primary cell lines. Vinculin and GAPDH served as loading controls. *E*, heatmap of log2 MS intensities for proteins unique to one condition (bulk or OCSCs). Adj.P.Val = Benjamini-Hochberg adjusted *p* value; PC = principal component.

The principal component analysis (PCA) of the proteome data showed a grouping of the samples according to culture condition on component-1, explaining 34.2% of the total variance (Fig. 2*A*). Component-2, which explained 21% of the variance, resolved the inter-cell line heterogeneity, which was more extreme for bulk than OCSCs.

We performed differential expression analysis to identify significantly regulated proteins between OCSCs and bulk cultures. We identified 595 differentially abundant proteins: 246 proteins were statistically more abundant in stem cells, while 349 were statistically more abundant in adherent cultures (Fig. 2*B* and supplemental Table S2). This set of proteins behaved consistently and homogeneously across the 10 patients (Fig. 2*C*) and from now on will be referred to as the “stemness signature”. The most significantly up-regulated protein in spheres was the Glycoprotein Nonmetastatic Melanoma Protein B (GPNMB; adjusted *p* value = 1.68E-07; Log2 fold-change = 4.73; supplemental Fig. S3*A*), which is a transmembrane glycoprotein highly expressed in multiple cancers (40). Additionally, the Chitinase-3 like Protein 1 (CHI3L1), which has also been implicated in several malignancy-associated cellular properties (41), was among the most significantly up-regulated proteins (adjusted *p* value = 1.75E-06; Log2 fold-change = 3.16; supplemental Fig. S3*B*). We further validated these hits by immunoblotting (Fig. 2*D*). Of note, the gene expression level of both GPNMB and CHI3L1 correlated with poor prognosis in HGSOC patients, being associated with shorter progression-free and overall survival, although the correlation of CHI3L1 with overall survival did not reach statistical significance (supplemental Fig. S4).

Examining proteins exclusively present in one condition and absent in the other, we identified 11 proteins unique to OCSCs and 34 unique to bulk cultures (Fig. 2*E*). Notably, among the proteins unique to OCSCs, we identified Matrix Gla protein (MGP) that we recently reported as a driver of stemness and tumor initiation in ovarian cancer (42).

**Fig. 3:**
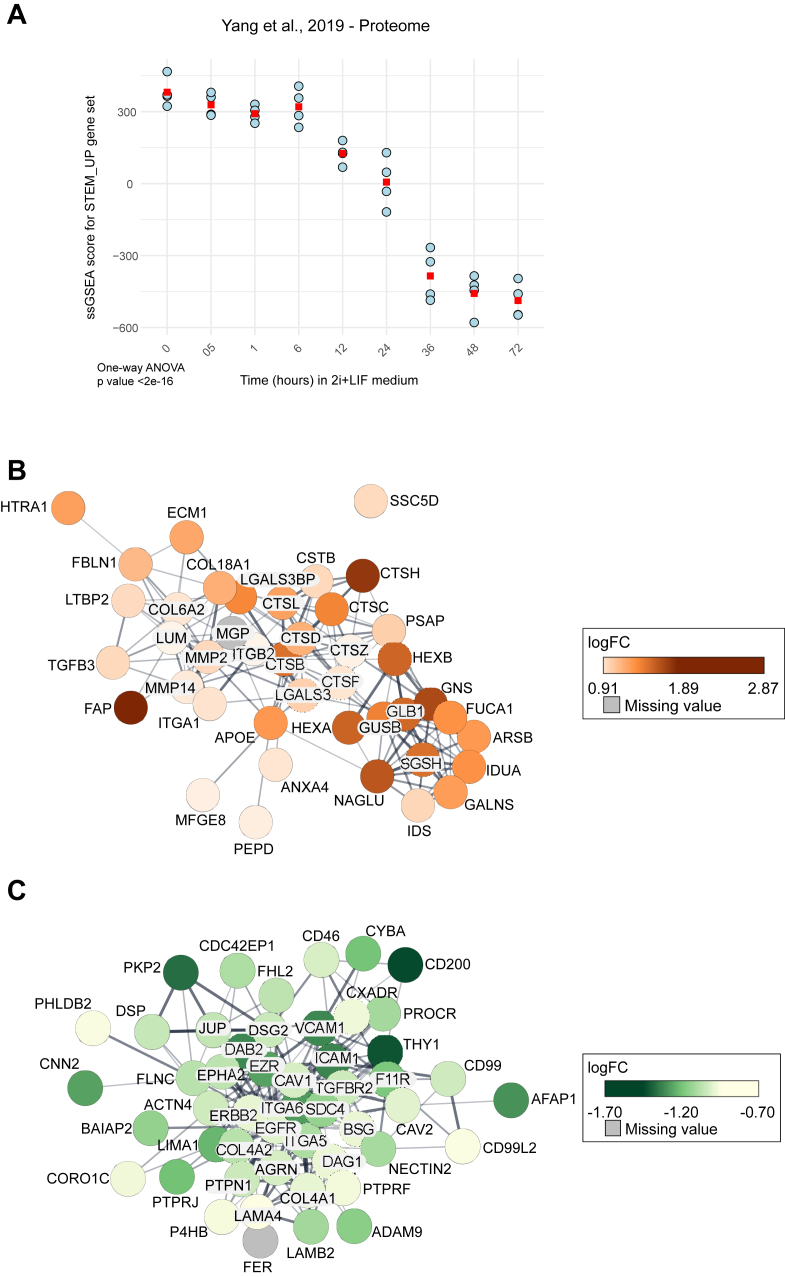
**Characterization of proteins altered by stemness**. *A*, single-sample gene set enrichment analysis (ssGSEA) of the proteome dataset from Yang *et al*. The gene set STEM_UP includes proteins significantly upregulated in OCSCs compared to bulk plus proteins unique to OCSCs. The squared dot in red represents the mean. The *p* value was calculated through one-way ANOVA. 2i = combination of MEK and GSK3 inhibition; LIF = leukemia inhibitory factor. *B* and *C*, functional STRING protein networks of proteins belonging to the gene ontology terms and pathways highlighted in *blue* in supplemental Figs. S7 and S8. Nodes with missing values correspond to proteins unique to OCSCs (*B*) or bulk (*C*).

To confirm that the above-described stemness signature was related to induction of stemness, we created two gene sets: one with proteins upregulated in OCSCs (referred to as “STEM_UP”) and the other with proteins upregulated in bulk cells (referred to as “STEM_DN”). Then, we performed single-sample GSEA (ssGSEA) on the proteomics data from Yang *et al*. (35), generated from embryonic stem cells transitioning from naive to primed pluripotency. A combined score considering both gene sets was also computed by default (referred to as “STEM”). This analysis showed the significant loss of the STEM and STEM_UP and the increase of the STEM_DOWN signatures over the time course (Fig. 3*A* and supplemental Fig. S5, *A* and *B*), validating our protein signature as a stemness signature. The best performing gene set in this analysis was the STEM_UP, showing the lowest one-way ANOVA *p* value (<2e-16).

Cultural conditions can deeply impact cell biology. Spheroids possess an inner apoptotic core (43). Moreover, a hallmark of stemness is quiescence (44). Therefore, to evaluate if the observed difference between OCSCs and bulk cells was not due to differential cell proliferation, we analyzed a set of genes known to correlate with cell proliferation rates (45). The median measurement of this set is referred to as “proliferation index”. We did not observe any significant difference in proliferation index between the spheroid and adherent condition (supplemental Fig. S6; paired *t* test, *p* value = 0.3904). However, we noticed that three adherent cell cultures (P6, P8, P9) displayed a higher index (one-way ANOVA in adherent group, *p* value = 0.0452). The same three samples were also outliers in the PCA on component-2 (Fig. 1*D*).

### Alteration of Signaling Pathways in OCSCs

Gene ontology (GO) over-representation analysis (ORA; supplemental Table S4) showed that adherent cells were enriched in plasma membrane proteins (supplemental Fig. S7), while OCSCs were enriched in lysosomal and extracellular proteins (supplemental Fig. S8). The same analysis indicated that processes related to cell adhesion were enriched in bulk cultures (Fig. 3*B* and supplemental Fig. S8), consistently with the lack of anchorage in spheroid cultures. Conversely, OCSCs were enriched in processes related to extracellular matrix (ECM) remodeling (Fig. 3*C* and supplemental Fig. S7). Consistently, GSEA, using the curated gene sets CGP (chemical and genetic perturbations; supplemental Table S5), highlighted that genes down-regulated in the epithelial-mesenchymal transition (EMT) upon transforming growth factor beta were enriched in adherent cells (supplemental Fig. S9*A*). Multiple EMT-associated markers (CDH1, CDH2, FN1, SNAI1, SNAI2, TWIST, VIM and ZEB1) were further validated by qRT-PCR (supplemental Fig. S9*B*). This further confirmed the stemness nature of the spheres, as EMT is commonly considered as a hallmark of CSCs (46). Spheres also appeared enriched in a wide variety of metabolic processes of both carbohydrates and lipids (supplemental Fig. S7). The increased metabolic output was accompanied by higher abundance of proteins involved in OxPhos, as shown by the enrichment of the gene set “oxidative phosphorylation” (supplemental Fig. S10*A*), obtained through GSEA using the hallmark gene sets (supplemental Table S5). Glycolysis was conversely upregulated in the adherent condition (supplemental Fig. S10*B*).

### Clustering Analysis Reveals Interpatient Heterogeneity in Stemness-Associated Changes

To characterize the heterogeneity in the biological processes and pathways contributing to stemness, we performed hierarchical clustering of the fold-changes OCSCs vs bulk for all proteins (supplemental Fig. S11*A*). Here, we identified two main clusters: cluster A, composed by patients 1, 2, 3, 4, 5 and 7; and cluster B, composed by patients 6, 8, 9 and 10. Proteins with fold-changes significantly different between the two patient clusters (Mann-Whitney *U* Test *p* value < 0.02) are shown in supplemental Fig. S11*B*. Set 1 includes proteins up-regulated in cluster A, and is enriched in processes related to membrane trafficking (supplemental Fig. S11*C*). Set 2 includes proteins with higher fold-change in cluster B and is enriched in processes related to mitochondria (supplemental Fig. S11*D*).

### Augmented Stemness Signature in HGSOC Tumors Refractory to Chemotherapy

**Fig. 4:**
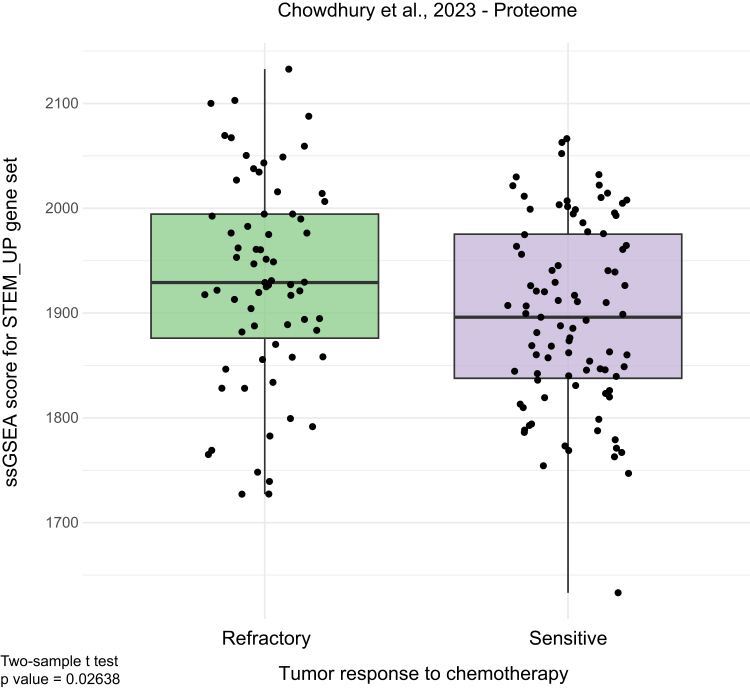
**Augmented stemness signature in HGSOC tumors refractory to chemotherapy**. Single-sample gene set enrichment analysis (ssGSEA) of the proteome dataset from Chowdhury *et al*. The gene set STEM_UP includes all proteins up-regulated in OCSCs compared to bulk plus proteins unique to OCSCs. The *p* value was calculated through unpaired, two-sided, two-sample Student's *t* test.

OCSCs have been implicated in unresponsiveness to chemotherapy (47, 48). Therefore, we asked if the stemness signature defined in the current study was associated with chemorefractory HGSOC. To test this hypothesis, we used the CPTAC proteome dataset from Chowdhury *et al*. (8) including 158 FFPE (formalin-fixed and paraffin embedded) refractory and sensitive HGSOC patient biopsies. With the gene sets derived from our study, we performed ssGSEA and showed that proteins upregulated in OCSCs (“STEM_UP” gene set) were significantly upregulated in chemorefractory tumors (two-sample *t* test, *p* value = 0.02638) (Fig. 4). As we observed significant differences between the different sample sources (one-way ANOVA, *p* value = 1.48e-09) (supplemental Fig. S12*A*), we also validated these findings by performing a significance test using the sample source as covariate (two-way ANOVA, *p* value = 0.0116) (supplemental Fig. S12*B*).

### Phosphoproteomics Reveals the Activation of PDGF Signaling in OCSCs

**Fig. 5:**
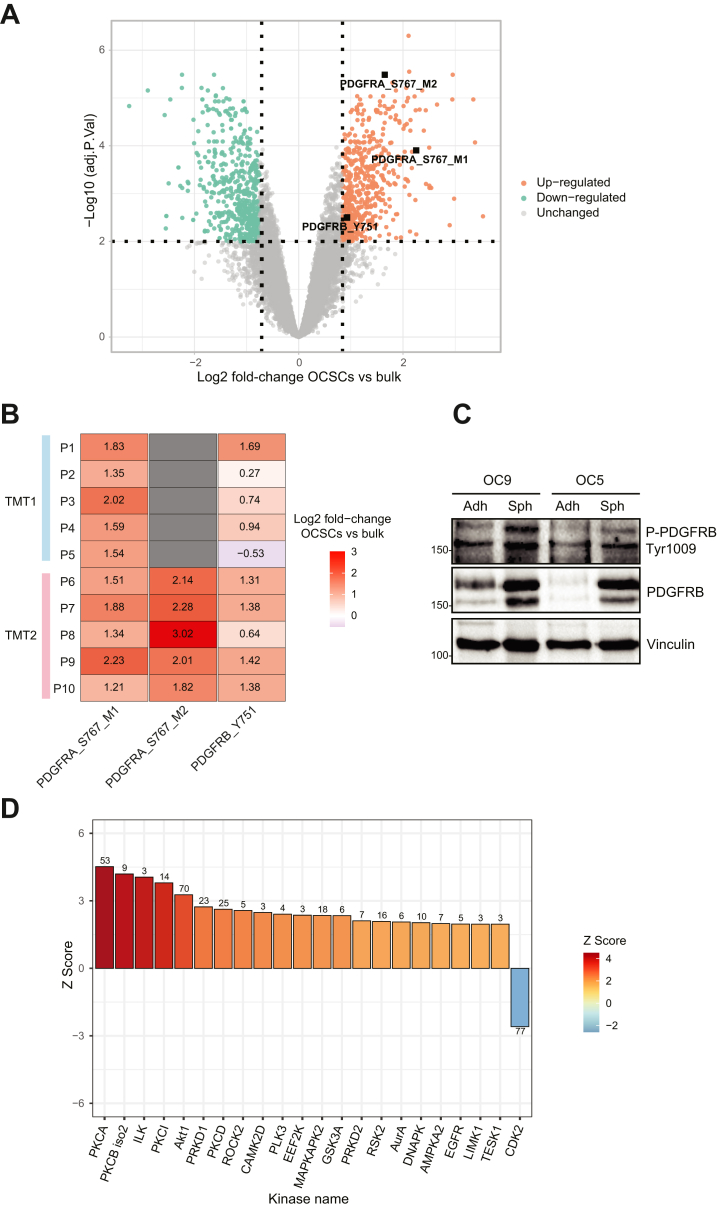
**Phosphoproteomic analysis of ovarian cancer stem cells**. *A*, Volcano plot analysis of the phosphoproteomic data. Phosphorylation sites colored in *orange* or *green* are considered up- or down-regulated, respectively. Significantly regulated phosphosites from the PDGF receptors are labeled. *B*, heatmap of log2 fold-changes in OCSCs vs bulk for each individual patient pair for the PDGFR sites highlighted in *A*. *C*, immunoblot analysis for phosphorylated and total PDGFRB in whole cell lysates from two primary ovarian cancer cell lines. *D*, RoKAI inference of kinase activity from the phosphoproteomic data. Kinases with a number of substrates ≥ 3 and *p* value ≤ 0.05 are shown.

Next, we performed PCA on the phosphoproteome data, and observed a clear separation between OCSCs and bulk (supplemental Fig. S13*A*), which remained virtually unchanged after normalization to protein quantities (supplemental Fig. S13*B*), indicating that normalization had minimal impact on the overall clustering. Additionally, we performed differential expression analysis to identify significantly regulated phosphosites between OCSCs and bulk cultures. We identified 1059 differentially abundant phosphosites: 519 were statistically more abundant in OCSCs, while 484 were statistically more abundant in bulk cultures (Fig. 5*A* and supplemental Table S3). Among the upregulated sites, we identified two sites from the platelet-derived growth factor receptors α and β (PDGFRA and B, respectively) (Fig. 5, *A* and *B*). PDGFRA protein was itself upregulated (*p* value = 4.84E-05; adjusted *p* value = 0.0007; Log2 fold-change = 0.71; supplemental Fig. S14*A*), but this did not appear to account for its increased phosphorylation status (supplemental Fig. S14*D*). PDGFRB protein was also upregulated but not significantly (*p* value = 0.1; adjusted *p* value = 0.19; Log2 fold-change = 0.47; supplemental Fig. S14*B*). However, when analyzing all precursors, its abundance appeared to be increased in all patients except number 8 (paired *t* test *p* value = 0.007; Log2 fold-change = 1.67; supplemental Fig. S14*C*), and could account for the higher phosphorylation observed in patients 2, 3, 4 and 5 (supplemental Fig. S14*D*). To confirm PDGFR upregulation, we analyzed PDGFRB abundance and its phosphorylation at tyrosine 1009, a different activation site not identified by MS, by immunoblotting in two patient-derived primary cell lines (number 5 and 9). This analysis showed consistent upregulation in OCSCs for both models (Fig. 5*C*). Next, we employed the RoKAI algorithm, which assesses kinase activity by analyzing alterations in the phosphorylation of known kinase substrates and their functional network (38). PDGFR was found to have increased activity in this analysis (Z score = 1.72; *p* value = 0.086) because of the increase in its autophosphorylation site at the tyrosine 751, which is located in the tyrosine kinase domain. Additionally, most of the kinases with increased activity in OCSCs (Fig. 5*D*) belonged to signaling cascades downstream of PDGFR. For example, upon activation by PDGF, PDGFR recruits and activates PI3K, leading to the generation of PIP3 and subsequent activation of AKT1 (49). PKC and PKD are also activated downstream of PDGFR through the phospholipase C gamma (PLCγ)/diacylglycerol (DAG) pathway (50). Rho-associated kinase 2 (ROCK2), involved in cytoskeletal rearrangement and cell migration, is regulated by the Rho family GTPase that is activated by PDGFR (51). PDGFR activation can also initiate the MAPK pathway (52), and MAPKAPK2 is downstream of p38 MAPK, a component of the MAPK pathway.

### Role of PDGF Receptor in Stemness

**Fig. 6:**
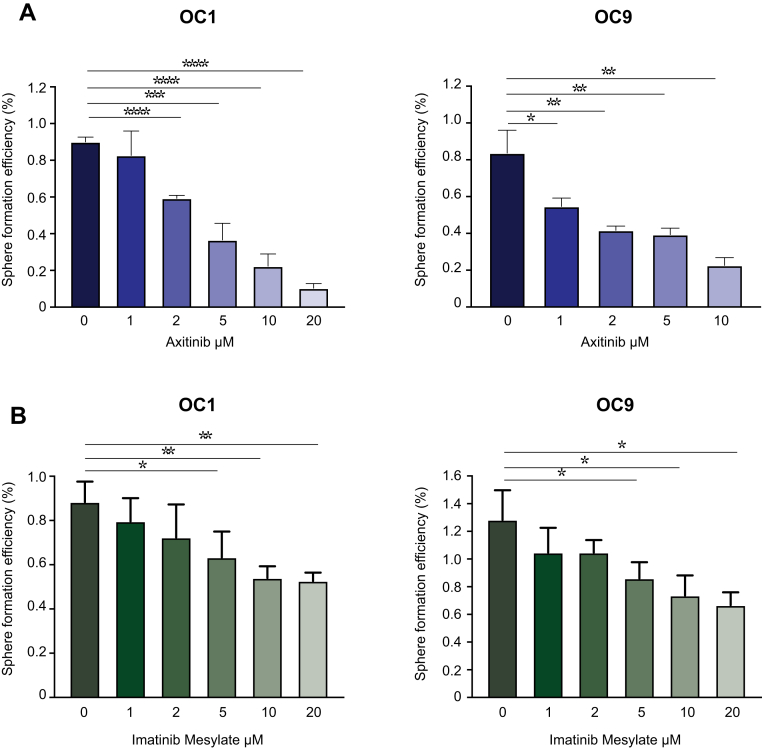
**PDGFR role in stemness**. Sphere formation assay of two primary ovarian cancer cell lines, after treatment with the PDGF receptor inhibitors Axitinib (*A*) and Imatinib Mesylate (*B*).

To investigate PDGFR role in HGSOC stem cells, we pharmacologically inhibited PDGFR in the cell lines derived from patients 1 and 9, using two different small-molecule tyrosine kinase inhibitors, Axitinib and Imatinib Mesylate, which are known to repress PDGFR signaling along with other kinases. Axitinib also inhibits VEGFR receptors (53), while Imatinib also targets v-Abl (54) and c-Kit (55). Both drugs demonstrated a significant, dose-dependent effect on sphere formation (Fig. 6, *A* and *B*), supporting the involvement of PDGFR in OCSCs survival and proliferation.

## Discussion

This study describes a detailed proteomic and phosphoproteomic analysis of HGSOC stem cells. By comparing patient-derived primary adherent (2D) cultures and spheroid (3D) counterparts, enriched for ovarian cancer stem cells (OCSCs), we identified significant molecular differences and pathways linked to stemness and chemoresistance.

The principal component analysis (PCA), on one hand, showed variability among different primary 2D cultures, which somehow reflects the well-known inter-patient heterogeneity. On the other hand, primary OCSCs from different patients tend to cluster together. This pattern suggests that, despite the intrinsic inter-individual variability, the molecular pathways associated with the stem-like phenotype of cancer cells are somehow conserved and shared across different patients. On this premise, it is conceivable that OCSC-targeted therapies could find broad applicability in HGSOC patients, possibly overcoming the heterogeneity of response to more conventional treatments, such as chemotherapy and PARP inhibitors.

Our approach proved suitable for identifying clinically relevant biomarkers, as demonstrated by the finding of GPNMB and CHI3L1 among the most upregulated proteins in OCSCs as compared to matched bulk cells. In fact, our analysis on a pool of publicly available datasets showed a significant correlation of both GPNMB and CHI3L1 gene expression with poor prognosis in HGSOC patients. This was in agreement with previous reports where high levels of the two proteins were found associated with unfavorable outcomes in ovarian cancer (56, 57). It is intriguing that CHI3L1 was proposed to promote ovarian cancer malignancy by enhancing tumor stemness (58), while GPNMB exerted an analogous function in breast cancer (59). Along the same line, one of the hits emerging from our proteome profiling of OCSCs was Matrix Gla Protein (MGP), which we have recently identified as a novel driver of OC stemness (42). Future studies should aim at validating GPNMB and CHI3L1 not only as markers of OCSC but also as causal players in OCSC-driven tumor progression and as putative targets in the context of tumor-eradicating strategies based on the elimination of the cancer stem cell subpopulation.

It has been reported that CSCs exhibit a unique metabolic phenotype compared to normal cancer cells. The latter metabolizes glucose to produce lactate through glycolysis even in the presence of sufficient oxygen (60), which is now known as the Warburg effect. Conversely, CSCs may be highly glycolytic or oxidative phosphorylation (OxPhos)-dependent depending on the niches where the CSCs are located (61). Here, we observed a metabolic switch in OCSCs, showing higher oxidative phosphorylation and lower glycolysis than bulk cells. This is in line with previous research in other tumor types (62, 63).

Previous research by Frederick *et al*. (64) used a method similar to ours to explore cellular signaling dynamics in EOC metastasis, where adherent cells model primary tumors and spheroid formation represents the early stages of metastatic spread. However, a key difference between their model and ours is that their spheroid formation protocol does not involve the selection of stem cells. While both models share common features, such as the upregulation of OxPhos, hypoxia and ROCK1 kinase, there are also significant differences. For instance, they found that spheroids enter a dormant state, while we observe no difference in cell proliferation markers. Additionally, they show increased glycolysis, while we observe a decrease in the glycolysis gene set. This further validates that our model is mainly driven by stemness rather than spheroid formation.

A limitation of our experimental approach stems from the limited protein amount per sample, which required us to use two different quantification methods: label-free quantification for the proteome and TMT labeling for the phosphoproteome. Consequently, we were unable to correctly normalize phosphorylation changes against protein quantities, making it difficult to distinguish whether the observed phosphorylation changes were due to alterations in protein levels.

An additional limitation of this work is the relatively small sample size (10 patient pairs), which restricted our ability to comprehensively analyze inter-patient variability. Although inter-patient heterogeneity is a hallmark of cancer biology, this study primarily aimed to identify conserved pathways and proteins associated with stemness by comparing OCSC-enriched populations and bulk ovarian cancer cells. To partially address inter-patient heterogeneity, we performed clustering of fold-change data to identify patterns of protein regulation that were consistent across patients. This approach revealed stemness-related pathways that exhibited some degree of patient group specificity. Future studies with larger cohorts will be essential to investigate patient-specific differences in greater detail and to further elucidate the impact of heterogeneity on stemness-related signaling.

Overall, our findings reveal that HGSOC CSCs possess unique proteomic and phosphoproteomic profiles, providing valuable insights for targeted therapies.

## Data Availability

Raw mass spectrometry files used in this experiment have been uploaded to the ProteomeXchange Consortium (http://proteomecentral.proteomexchange.org) *via* the PRIDE partner repository (65) with the dataset identifier PXD061015.

All the scripts utilized for data analysis and plotting are available at the following GitHub repository: https://github.com/Giu-F/OC_project.

Annotated spectra to support the identification of phosphorylated peptides from the TMT phosphoproteome data can be accessed through this link (https://msviewer.ucsf.edu/cgi-bin/mssearch.cgi?report_title=MS-Viewer&search_key=f0rfsl9krn&search_name=msviewer).

## Informed consent statement

Informed consent was obtained from all patients. Sample collection was performed under the protocol no. R789-IEO approved by the Ethics Committee of the European Institute of Oncology.

## Supplemental data

This article contains supplemental data.

## Conflict of interest

The authors declare that they have no conflicts of interest with the contents of this article.

## Supplementary Data

Supplementary table 1 Clinicopathological features of patients.

Supplementary table 2 Proteome data with statistical analysis.

Supplementary table 3 Phosphoproteome data with statistical analysis.

Supplementary table 4 GO, KEGG and Reactome enrichment.

Supplementary table 5 GSEA.

Supplementary table 6 RoKAI.

Supplementary table 7 TMT labeling scheme.

Supplementary table 8 Gene sets used for ssGSEA.

Supplementary table 9 Proteins used for STRING networks.

Supplementary table 10 GO, KEGG and Reactome enrichment for patient clusters.

Supplemental Figures Supplementary Figure 1. *A*, number of protein groups identified and quantified per sample in the proteomic data. *B*, number of phosphorylation sites identified and quantified per TMT plex in the phosphoproteomic data. *C*, localized phosphosites' overlap between TMT plexes. Supplementary Figure 2. *A*, patient-derived adherent cells or spheres from patient 13 were stained for the indicated markers (*red*) and counterstained with DAPI (*blue*). *B*, Patient-derived spheres from patient 14 were stained for the indicated markers (*red*) and counterstained with DAPI (*blue*). Scale bar: 50 μM. Supplementary Figure 3. Log2 MS intensities for the proteins GPNMB (*A*) and CHI3L1 (*B*). Supplementary Figure 4. Kaplan-Meier survival curves for the proteins GPNMB (*B* and *D*) and CHI3L1 (*A* and *C*), showing both overall survival (OS, upper graphs) and relapse-free survival (RFS, lower graphs). Supplementary Figure 5. Single-sample gene set enrichment analysis (ssGSEA) analysis of the proteome dataset from Yang et al. The gene set STEM (*A*) combines the gene sets STEM_UP (Fig. 3) and STEM_DN (*B*), which includes all proteins down-regulated in OCSCs compared to bulk plus protein unique to bulk. p values were calculated through one-way ANOVA. The squared dot in red represents the mean. 2i = combination of MEK and GSK3 inhibition; LIF, leukemia inhibitory factor. Supplementary Figure 6. Log2 MS intensities of 157 proteins associated with cell proliferation. Supplementary Figure 7. Gene ontology (*A* and *B*) and pathway (*C* and *D*) over-representation analysis (ORA) for proteins significantly upregulated in bulk cultures compared to OCSCs. The enrichment score is calculated as the number of significantly regulated proteins annotated with a term / number of proteins in the background that are annotated with the same term. Adj.P.Val = Benjamini-Hochberg adjusted p value. Supplementary Figure 8. Gene ontology (*A* and *B*) and pathway (*C* and *D*) over-representation analysis (ORA) for proteins significantly upregulated in OCSCs compared to bulk cultures. The enrichment score is calculated as the number of significantly regulated proteins annotated with a term / number of proteins in the background that are annotated with the same term. Adj.P.Val = Benjamini-Hochberg adjusted *p* value. Supplementary Figure 9. *A*, gene set enrichment analysis (GSEA) using the curated gene sets CGP (chemical and genetic perturbations). Proteins were ranked by log2 fold-change OCSCs vs bulk. *B*, mRNA expression of EMT-associated genes in OCSCs normalized to their adherent counterparts (*dashed lines*) in two patient-derived cell cultures. Comparisons between experimental groups were performed by unpaired, two-sided, two-sample Student's t test. ∗ = *p* < 0.05; ∗∗ = *p* < 0.01; ∗∗∗ = *p* < 0.005. Supplementary Figure 10. Gene set enrichment analysis (GSEA) using the hallmark gene sets for oxidative phosphorylation (*A*) and glycolysis (*B*). Proteins were ranked by Log2 fold-change OCSCs vs bulk. Supplementary Figure 11. *A*, Heatmap of log2 fold-changes OCSCs vs bulk for all proteins. Rows and columns were clustered using euclidian distance. *B*, Heatmap of log2 fold-changes OCSCs vs bulk for proteins significantly different from between the two patient clusters (Mann Whitney u test p value < 0.02). Rows were clustered using Pearson correlation, while columns using euclidian distance. *C* and *D*, pathway over-representation analysis (ORA) for proteins belonging to set 1 and 2. Supplementary Figure 12. Single-sample gene set enrichment analysis (ssGSEA) analysis of the proteome dataset from Chowdhury et al. The gene set STEM_UP includes all proteins up-regulated in OCSCs compared to bulk plus proteins unique to OCSCs. Samples in *A* are grouped by sample source. Samples in *B* are grouped by sample source and response to chemotherapy. *p* values were calculated by one-way (*A*) and two-way (*B*) ANOVA, respectively. Supplementary Figure 13. Principal component analysis (PCA) of the phosphoproteomic data without (*A*) or with (*B*) normalization on protein levels. Supplementary Figure 14. Log2 MS intensity (protein abundance) from the proteome data for PDGFRA (*A*) and PDGFRB (*B* and *C*). In *C*, protein quantification was performed by summing all PDGFR precursors. *D*, Phosphosite intensities were normalized on protein levels. The heatmap shows the log2 fold-changes OCSCs vs bulk for each individual patient pair for the sites displayed in Figure 5, *A* and *B*.
