# Supplementary material for: Quantitative Proteomics and Phosphoproteomics Analysis of Patient-Derived Ovarian Cancer Stem Cells
Source: Mol Cell Proteomics. 2025 Apr 7;24(5):100965. doi: 10.1016/j.mcpro.2025.100965 (PMC12142526; doi:10.1016/j.mcpro.2025.100965)
Supplement: Supplemental Figures — Supplementary Figure 1. A, number of protein groups identified and quantified per sample in the proteomic data. B, number of phosphorylation sites identified and quantified per TMT plex in the phosphoproteomic data. C, localized phosphosites' overlap between TMT plexes. Supplementary Figure 2. A, patient-derived adherent cells or spheres from patient 13 were stained for the indicated markers (red) and counterstained with DAPI (blue). B, Patient-derived spheres from patient 14 were stained for the indicated markers (red) and counterstained with DAPI (blue). Scale bar: 50 μM. Supplementary Figure 3. Log2 MS intensities for the proteins GPNMB (A) and CHI3L1 (B). Supplementary Figure 4. Kaplan-Meier survival curves for the proteins GPNMB (B and D) and CHI3L1 (A and C), showing both overall survival (OS, upper graphs) and relapse-free survival (RFS, lower graphs). Supplementary Figure 5. Single-sample gene set enrichment analysis (ssGSEA) analysis of the proteome dataset from Yang et al. The gene set STEM (A) combines the gene sets STEM_UP (Fig. 3) and STEM_DN (B), which includes all proteins down-regulated in OCSCs compared to bulk plus protein unique to bulk. p values were calculated through one-way ANOVA. The squared dot in red represents the mean. 2i = combination of MEK and GSK3 inhibition; LIF, leukemia inhibitory factor. Supplementary Figure 6. Log2 MS intensities of 157 proteins associated with cell proliferation. Supplementary Figure 7. Gene ontology (A and B) and pathway (C and D) over-representation analysis (ORA) for proteins significantly upregulated in bulk cultures compared to OCSCs. The enrichment score is calculated as the number of significantly regulated proteins annotated with a term / number of proteins in the background that are annotated with the same term. Adj.P.Val = Benjamini-Hochberg adjusted p value. Supplementary Figure 8. Gene ontology (A and B) and pathway (C and D) over-representation analysis (ORA) for proteins significantly upregulated i [file mmc11.pdf]

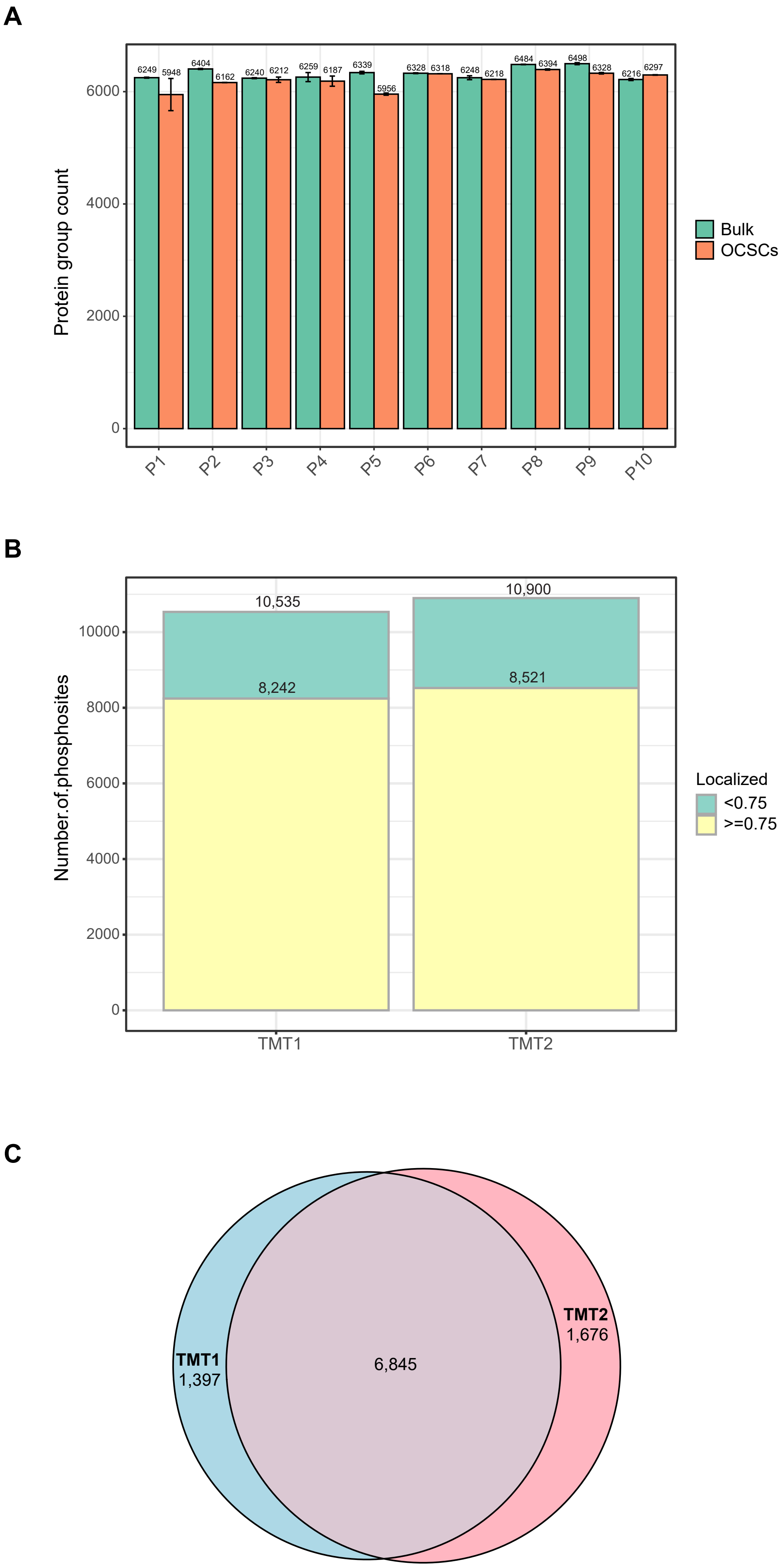

Supplementary figure 1

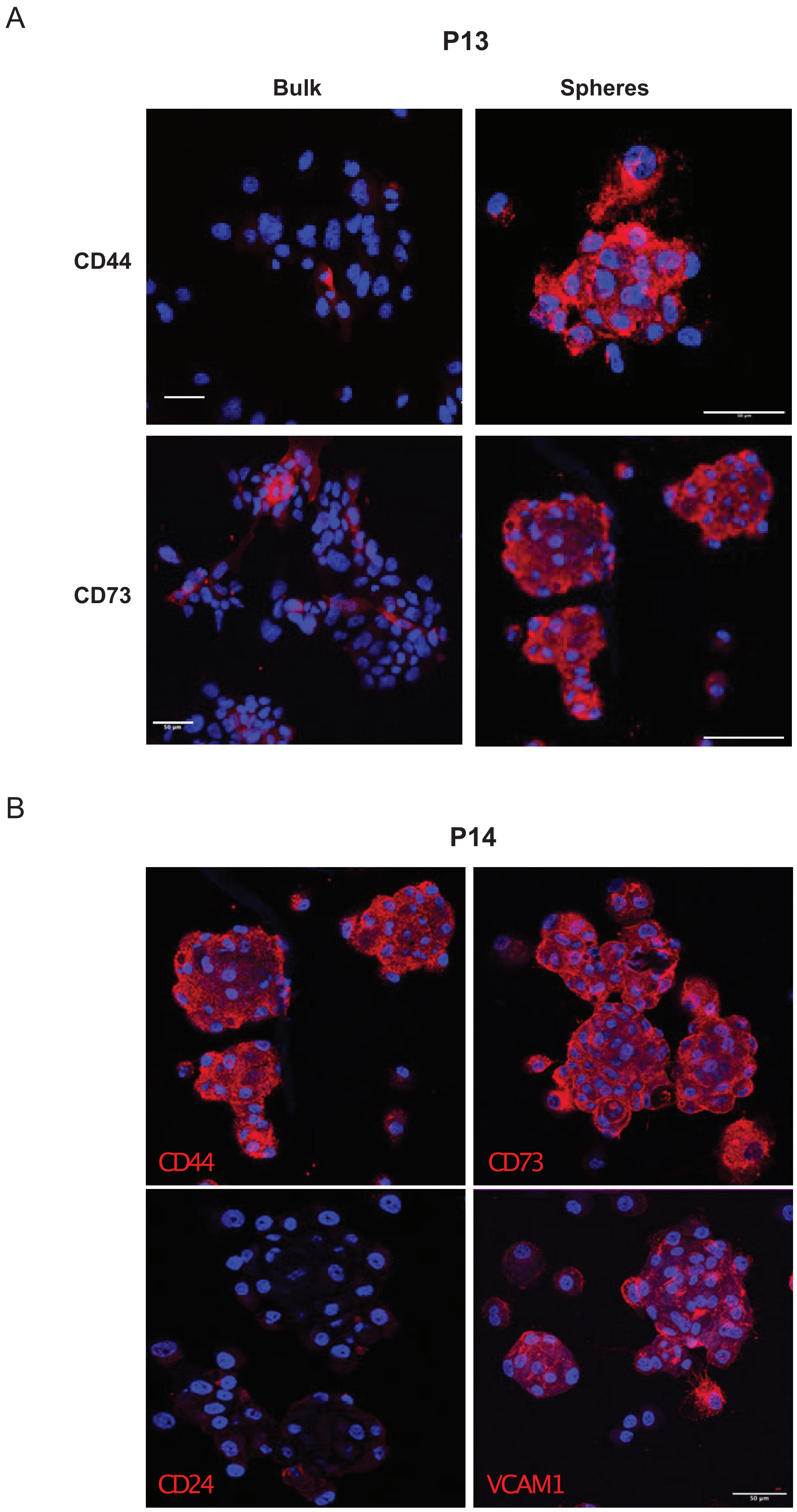

Supplementary figure 2

A

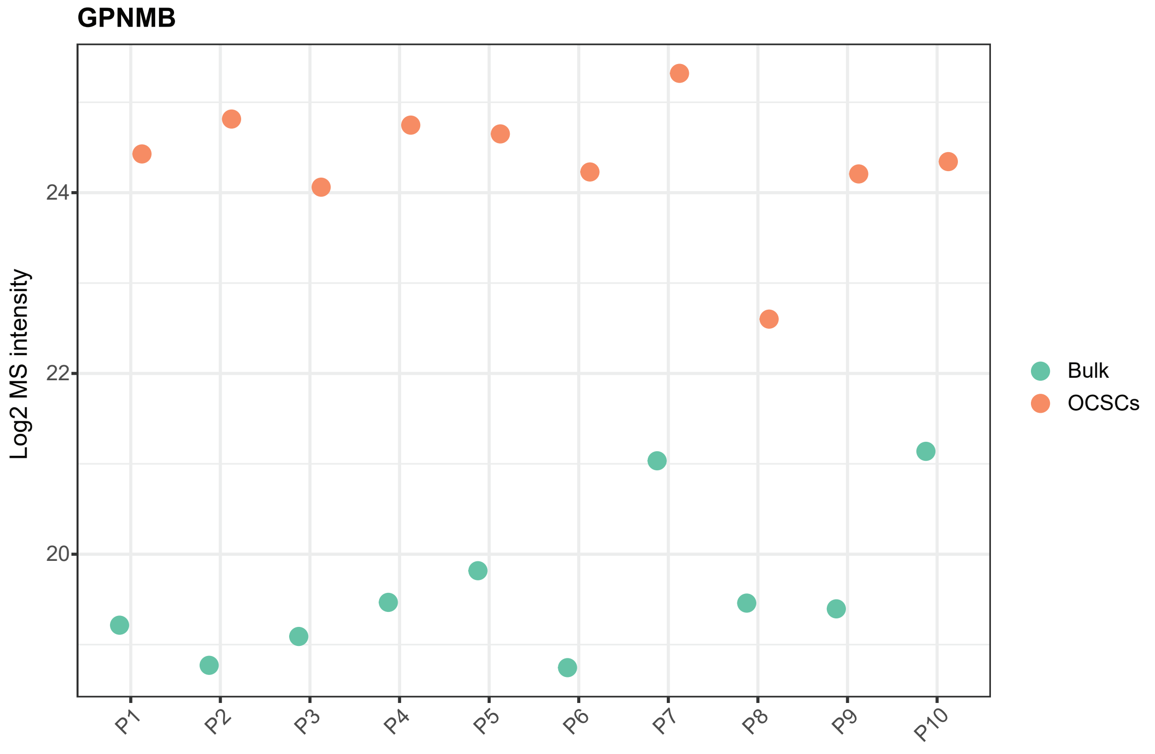

B

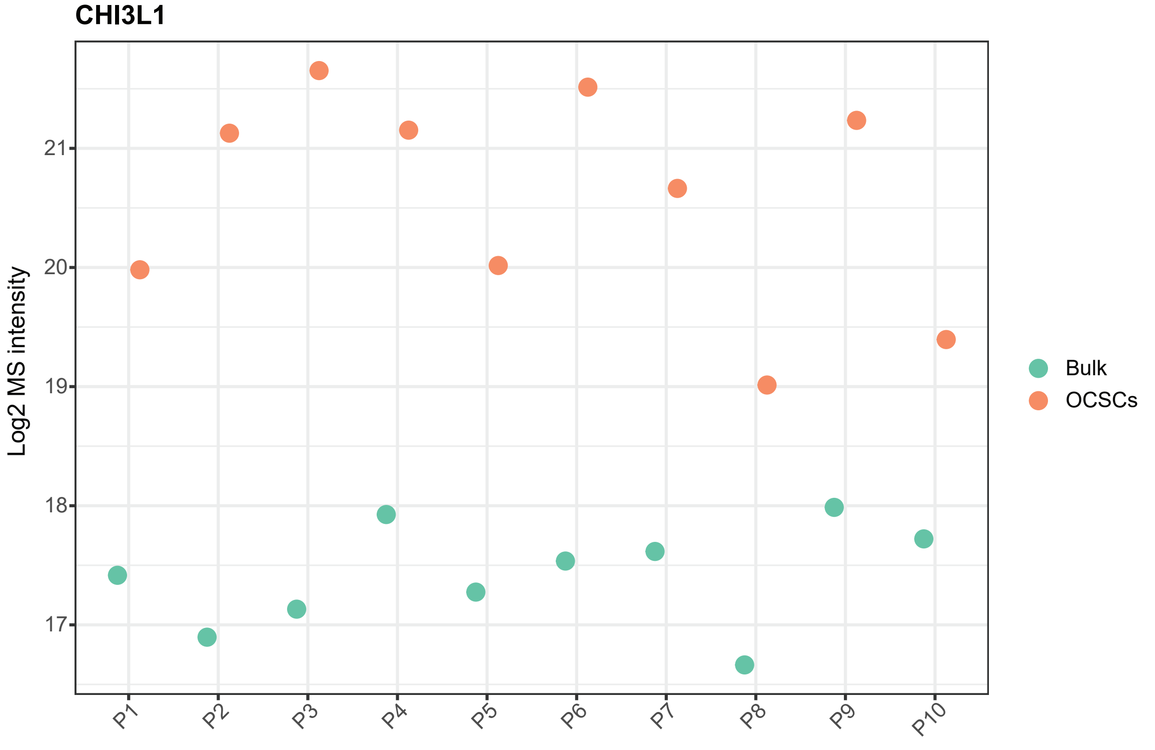

Supplementary figure 3

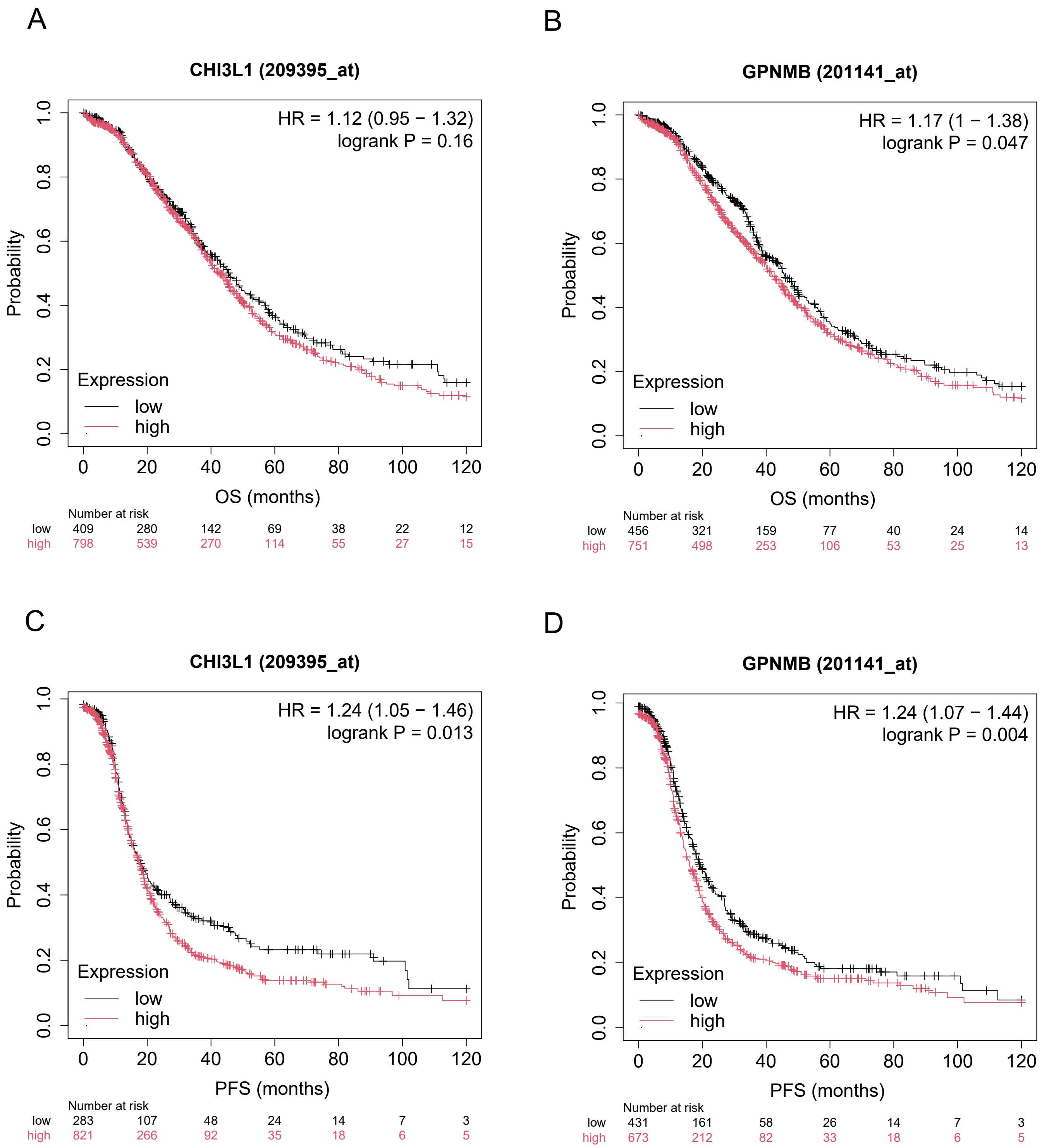

Supplementary figure 4

A

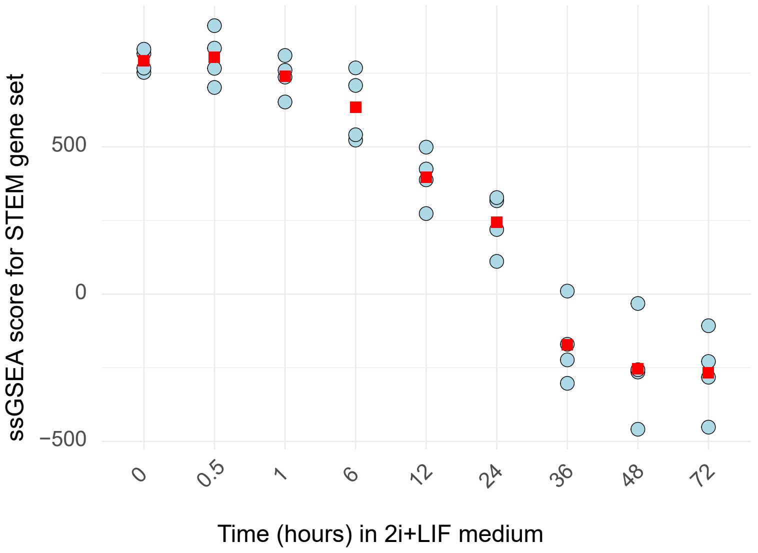

One-way ANOVA
p value 1.45e-15

B

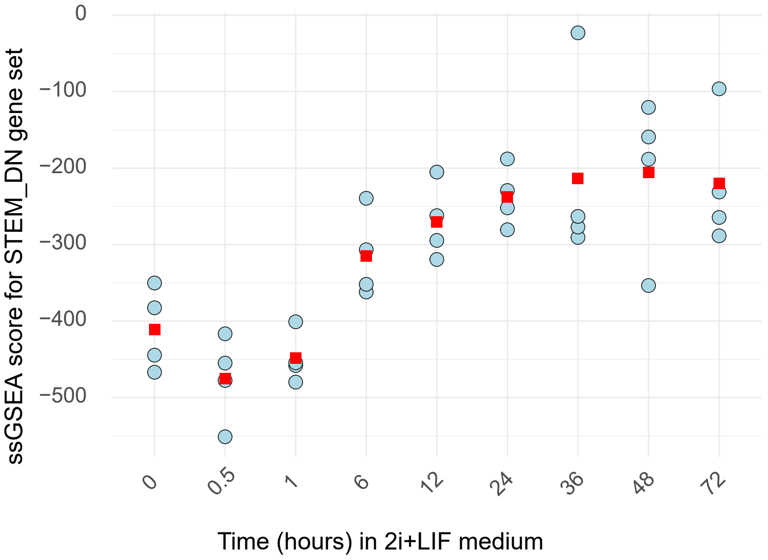

One-way ANOVA
p value 1.01e-05

Supplementary figure 5

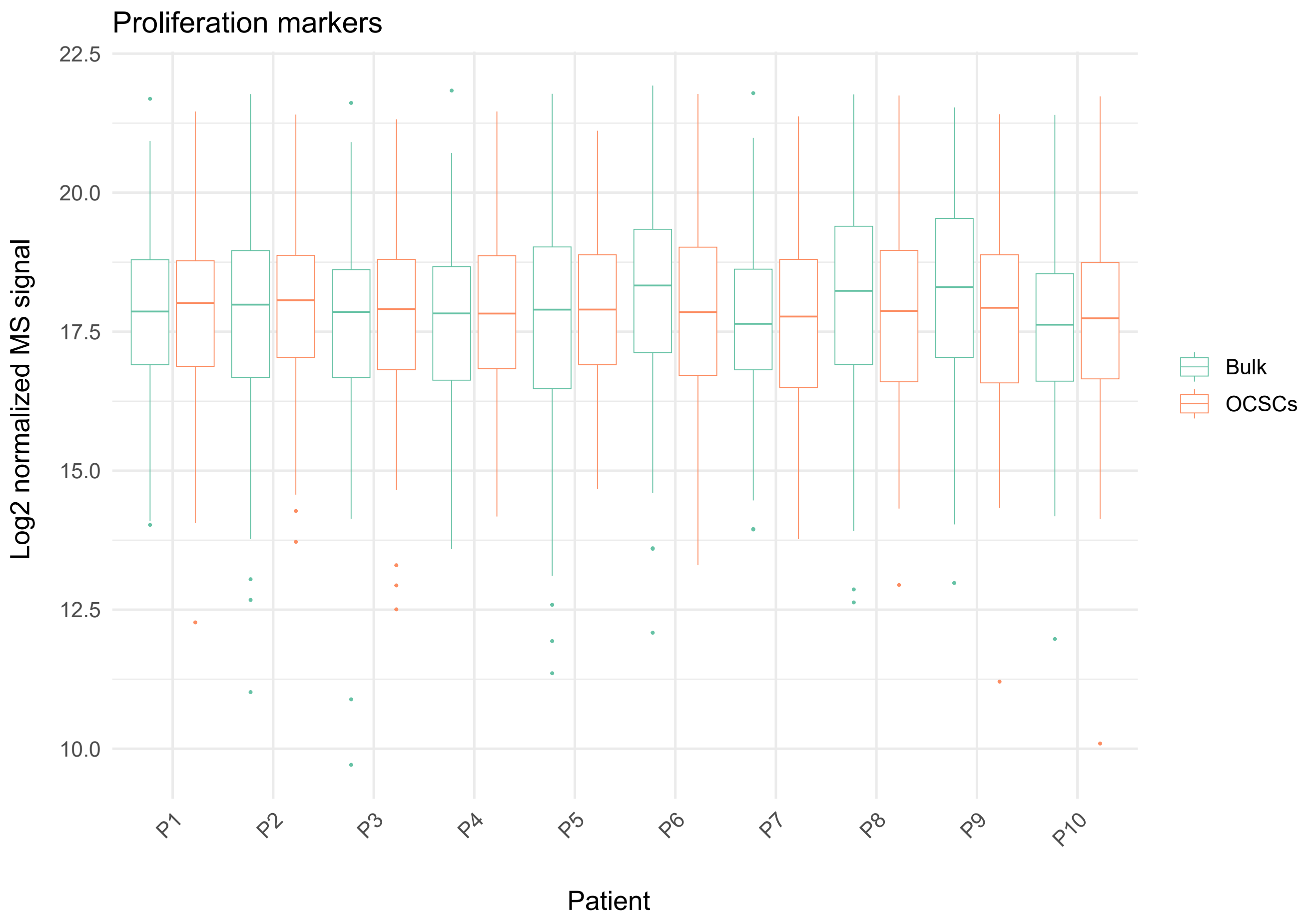

Supplementary figure 6

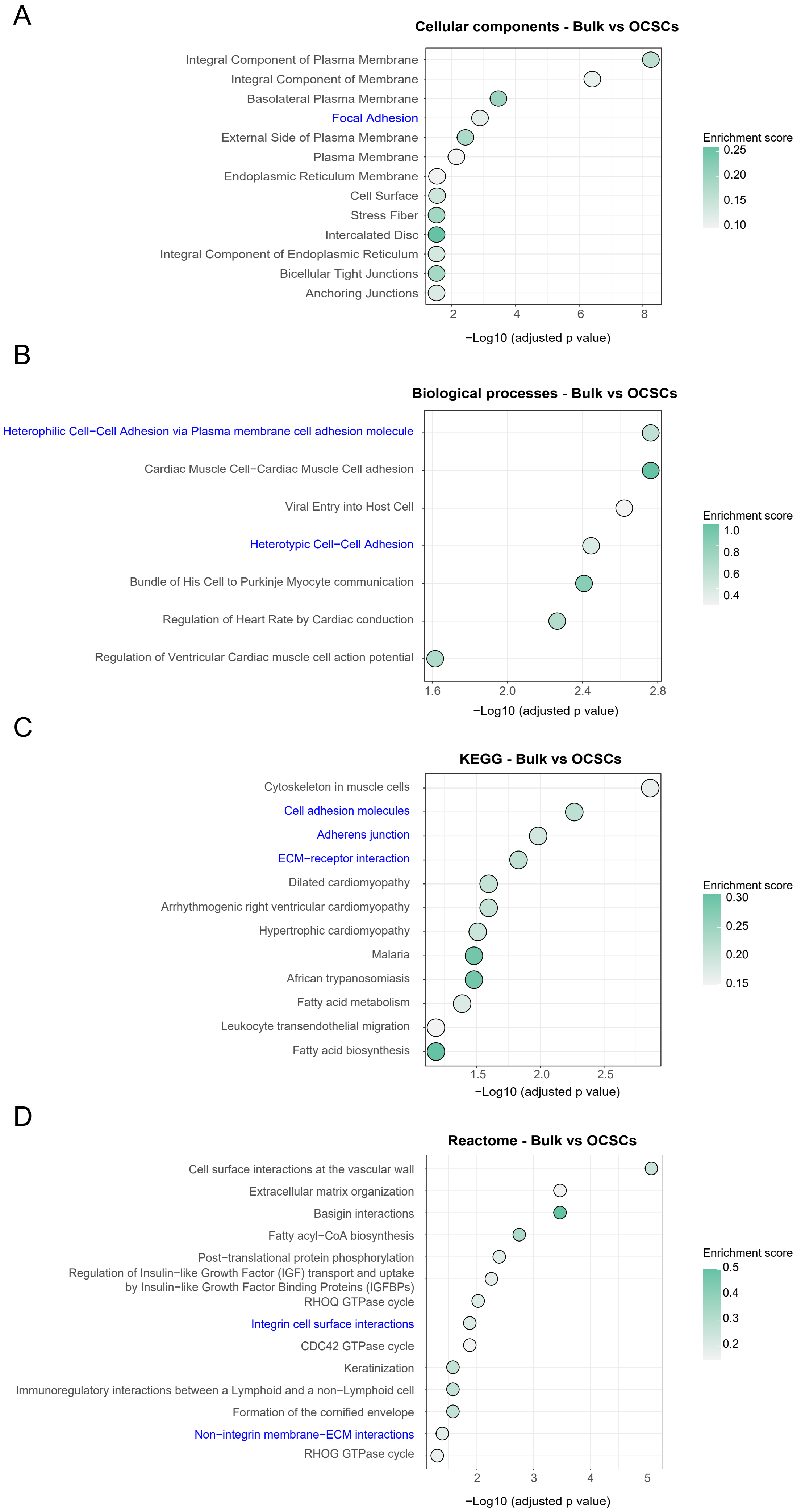

Supplementary figure 7

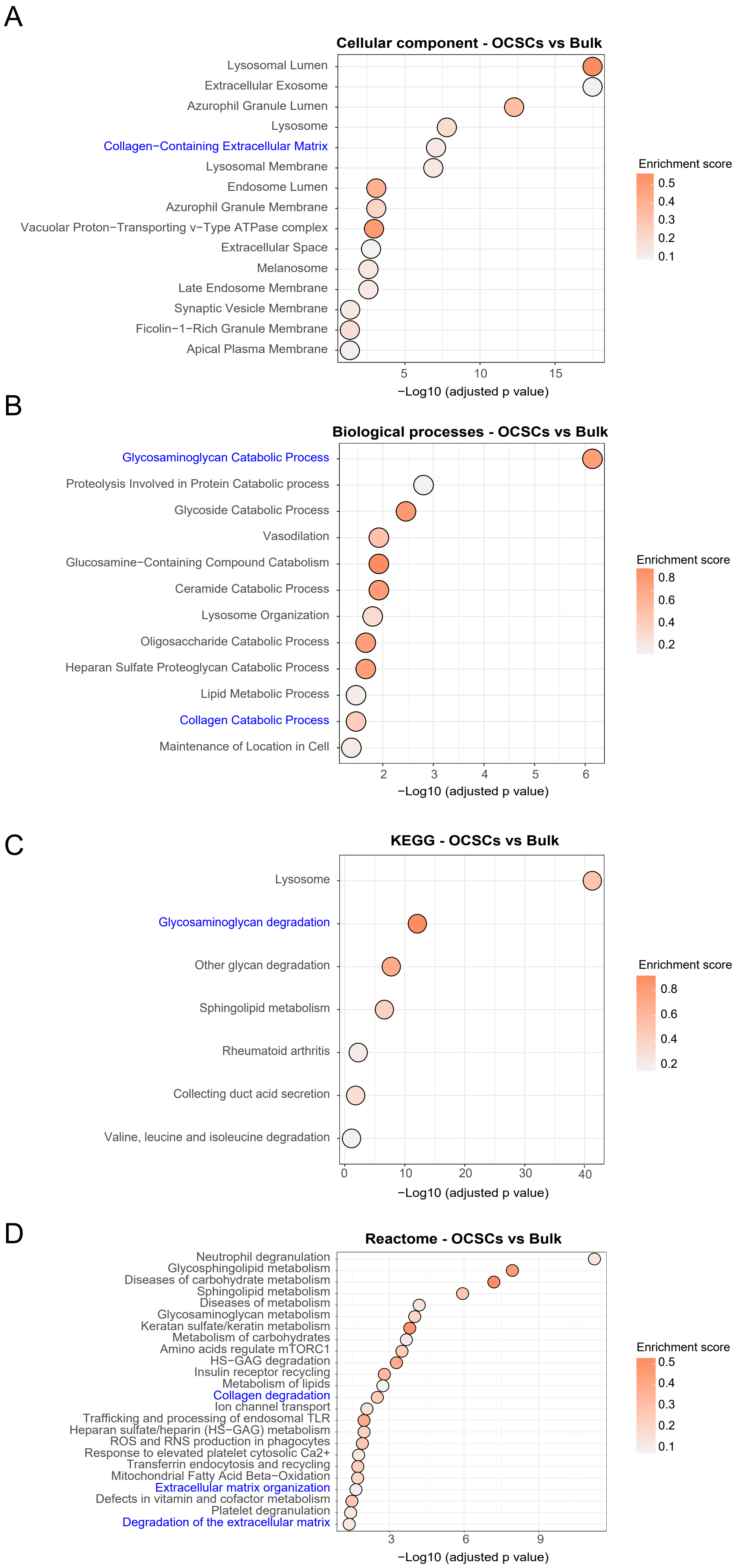

Supplementary figure 8

A

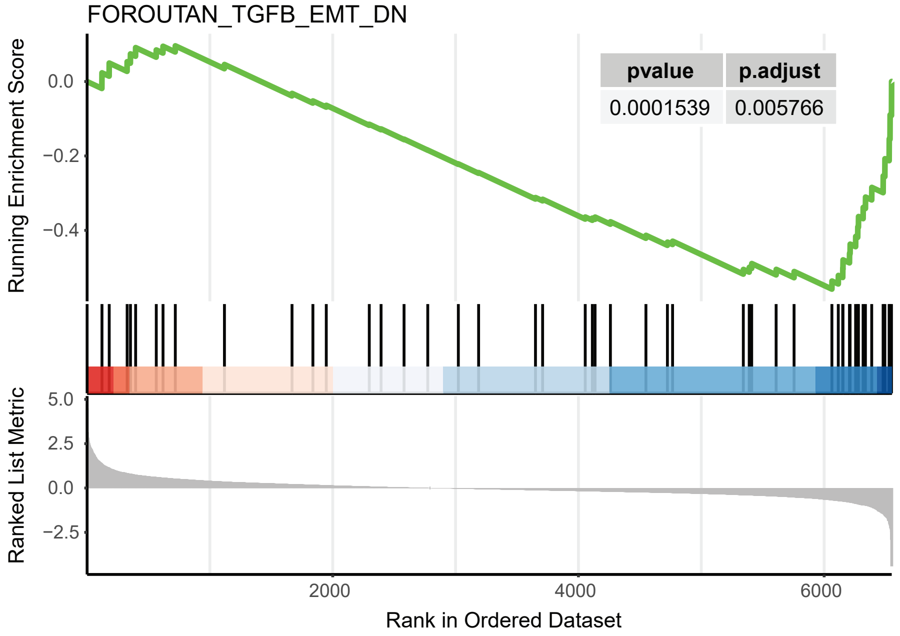

B

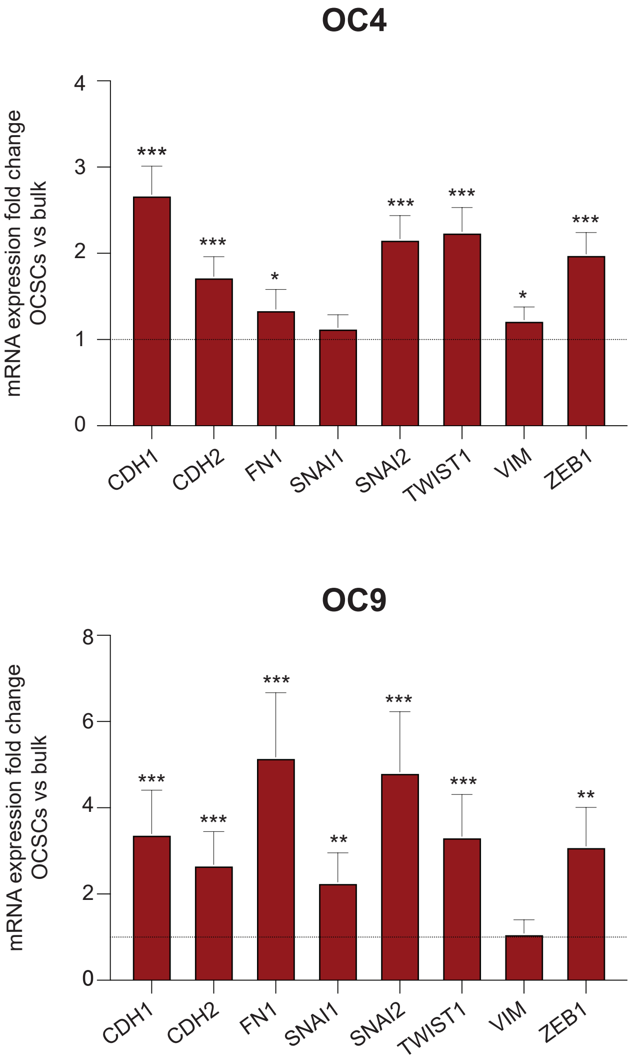

Supplementary figure 9

A

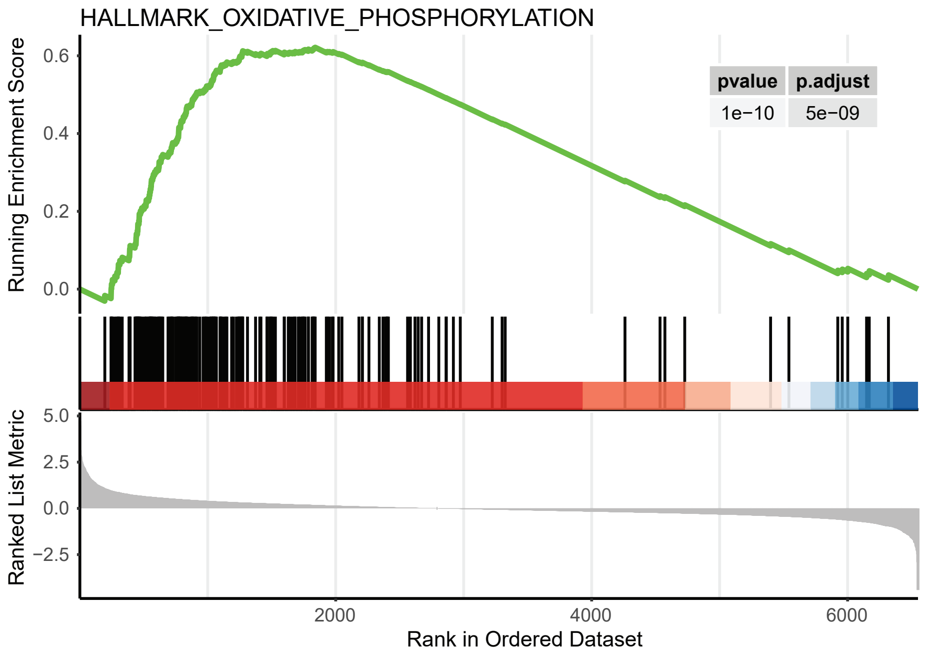

B

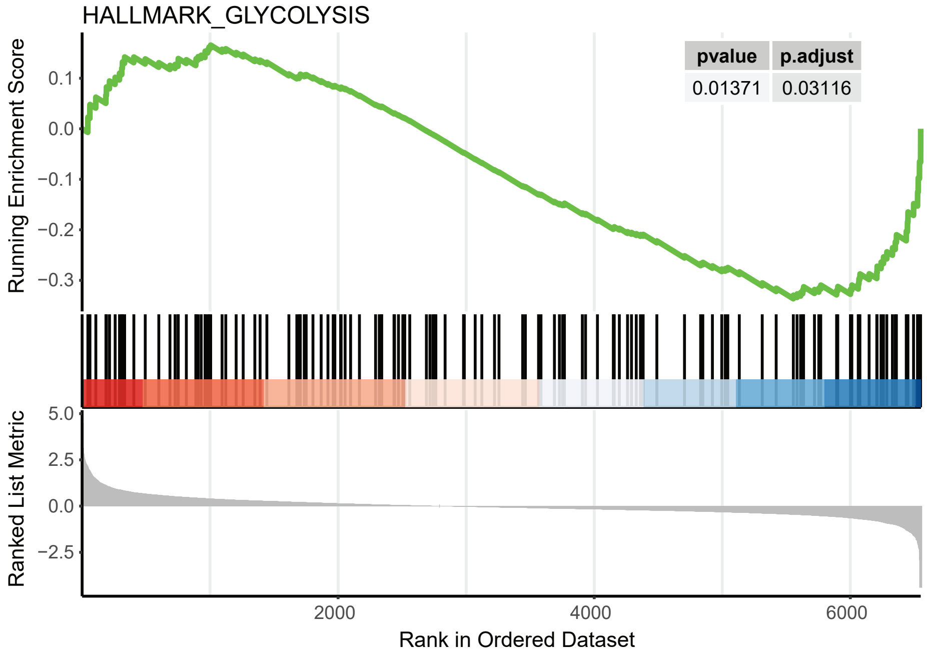

Supplementary figure 10

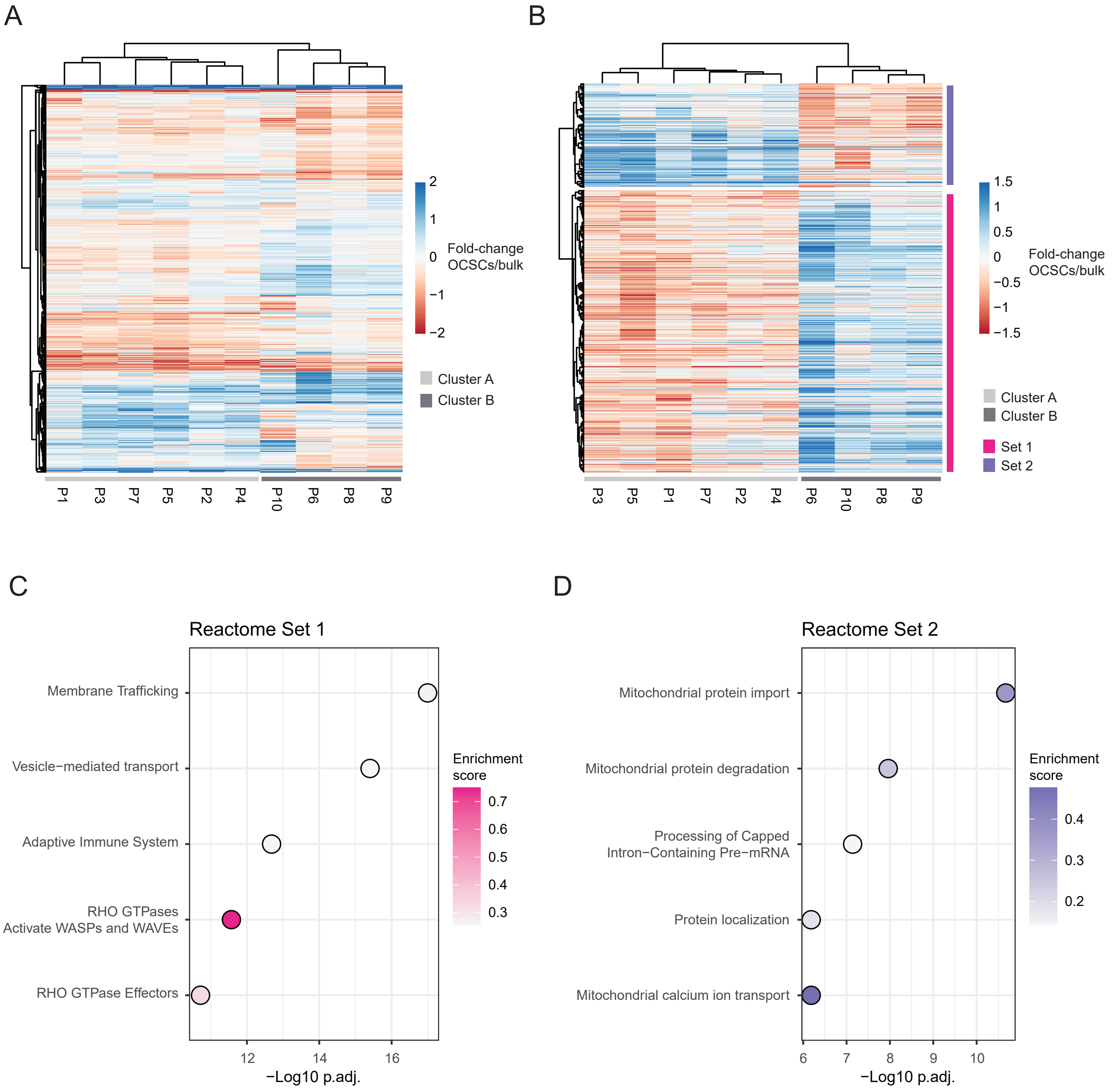

Supplementary figure 11

A

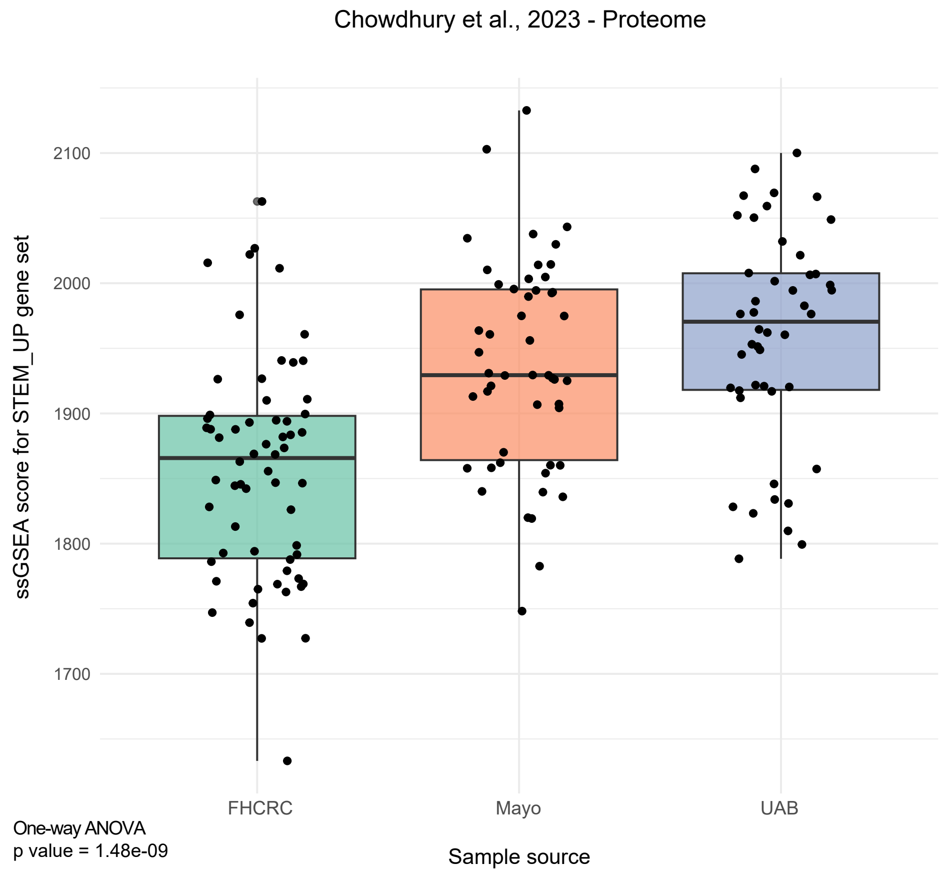

B

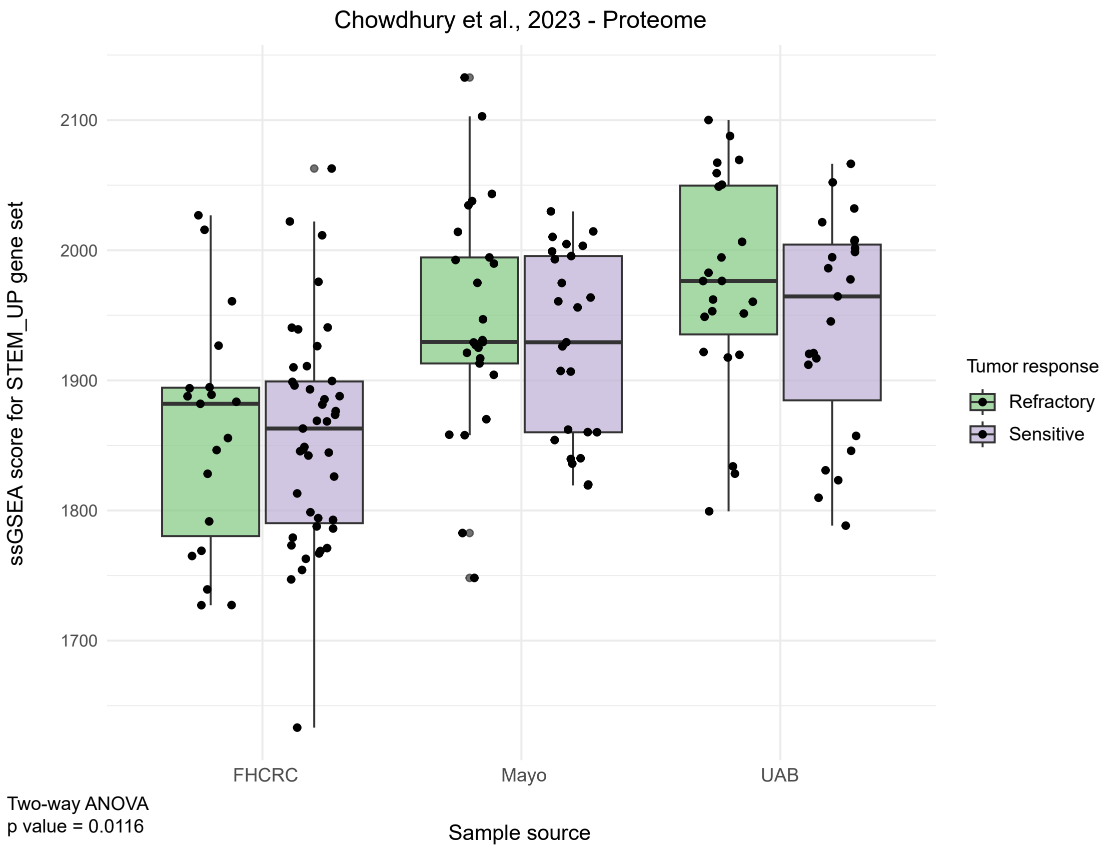

Supplementary figure 12

A

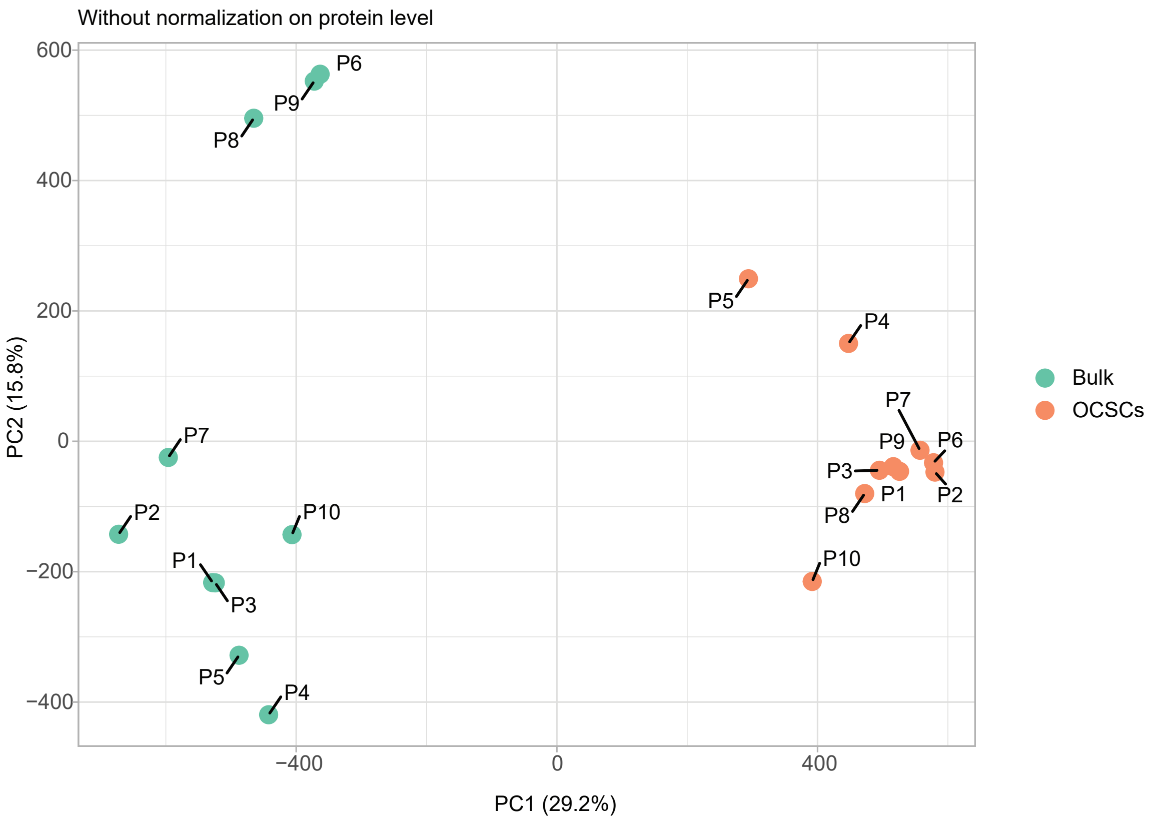

B

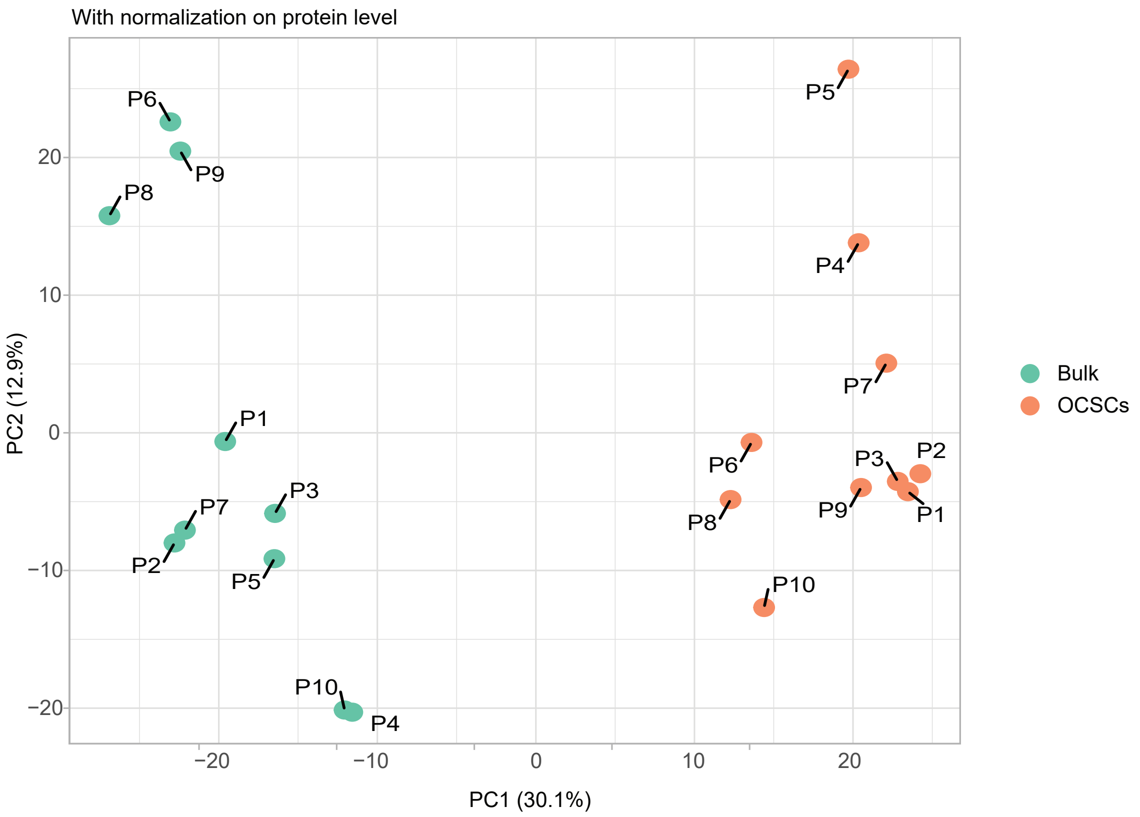

Supplementary figure 13

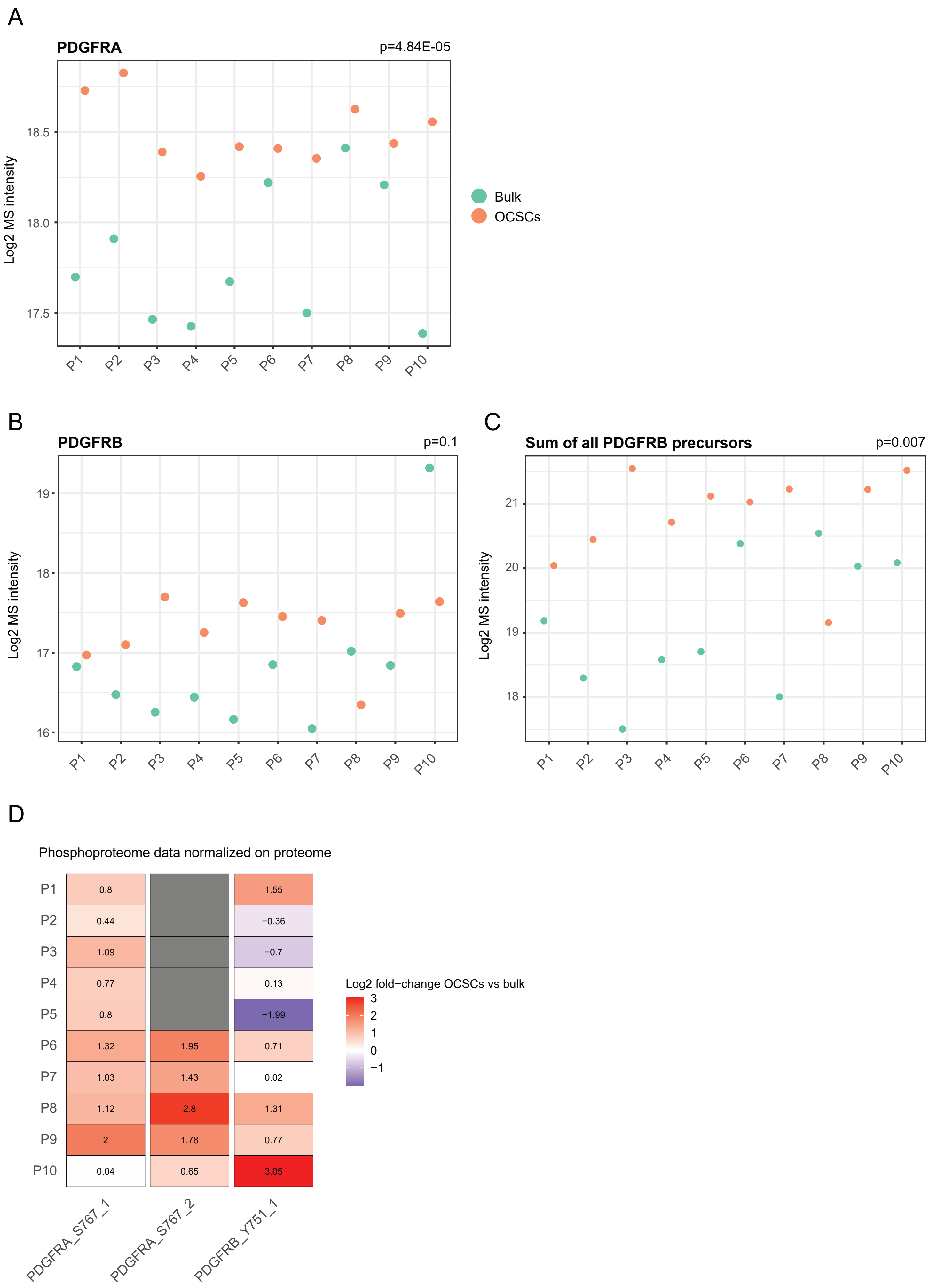

Supplementary figure 14
